# A unique mRNA decapping complex in trypanosomes

**DOI:** 10.1093/nar/gkad497

**Published:** 2023-06-13

**Authors:** Susanne Kramer, Natalia Katarzyna Karolak, Johanna Odenwald, Bernardo Gabiatti, Paula Andrea Castañeda Londoño, Anna Zavřelová, Eden Ribeiro Freire, Kayo Schemiko Almeida, Silke Braune, Claudia Moreira, Amelie Eder, Carina Goos, Mark Field, Mark Carrington, Fabiola Holetz, Maria Wiktoria Górna, Martin Zoltner

**Affiliations:** Biocenter, University of Würzburg, Würzburg, Germany; Biological and Chemical Research Centre, Department of Chemistry, University of Warsaw, Warsaw, Poland; Nencki Institute of Experimental Biology, Polish Academy of Sciences, Warsaw, Poland; Biocenter, University of Würzburg, Würzburg, Germany; Biocenter, University of Würzburg, Würzburg, Germany; Carlos Chagas Institute (ICC), FIOCRUZ/PR, Curitiba, Brazil; Biocenter, University of Würzburg, Würzburg, Germany; Department of Parasitology, Faculty of Science, Charles University in Prague, Biocev, Vestec, Czech Republic; Carlos Chagas Institute (ICC), FIOCRUZ/PR, Curitiba, Brazil; Carlos Chagas Institute (ICC), FIOCRUZ/PR, Curitiba, Brazil; Biocenter, University of Würzburg, Würzburg, Germany; Biocenter, University of Würzburg, Würzburg, Germany; Carlos Chagas Institute (ICC), FIOCRUZ/PR, Curitiba, Brazil; Biocenter, University of Würzburg, Würzburg, Germany; Biocenter, University of Würzburg, Würzburg, Germany; School of Life Sciences, University of Dundee, Dundee, UK; Biology Centre, Czech Academy of Sciences, Institute of Parasitology, České Budějovice, Czech Republic; Department of Biochemistry, University of Cambridge, Cambridge, UK; Carlos Chagas Institute (ICC), FIOCRUZ/PR, Curitiba, Brazil; Biological and Chemical Research Centre, Department of Chemistry, University of Warsaw, Warsaw, Poland; Department of Parasitology, Faculty of Science, Charles University in Prague, Biocev, Vestec, Czech Republic

## Abstract

Removal of the mRNA 5′ cap primes transcripts for degradation and is central for regulating gene expression in eukaryotes. The canonical decapping enzyme Dcp2 is stringently controlled by assembly into a dynamic multi-protein complex together with the 5′-3′exoribonuclease Xrn1. Kinetoplastida lack Dcp2 orthologues but instead rely on the ApaH-like phosphatase ALPH1 for decapping. ALPH1 is composed of a catalytic domain flanked by C- and N-terminal extensions. We show that *T. brucei* ALPH1 is dimeric *in vitro* and functions within a complex composed of the trypanosome Xrn1 ortholog XRNA and four proteins unique to Kinetoplastida, including two RNA-binding proteins and a CMGC-family protein kinase. All ALPH1-associated proteins share a unique and dynamic localization to a structure at the posterior pole of the cell, anterior to the microtubule plus ends. XRNA affinity capture in *T. cruzi* recapitulates this interaction network. The ALPH1 N-terminus is not required for viability in culture, but essential for posterior pole localization. The C-terminus, in contrast, is required for localization to all RNA granule types, as well as for dimerization and interactions with XRNA and the CMGC kinase, suggesting possible regulatory mechanisms. Most significantly, the trypanosome decapping complex has a unique composition, differentiating the process from opisthokonts.

## Graphical Abstract

**Graphical Abstract:**
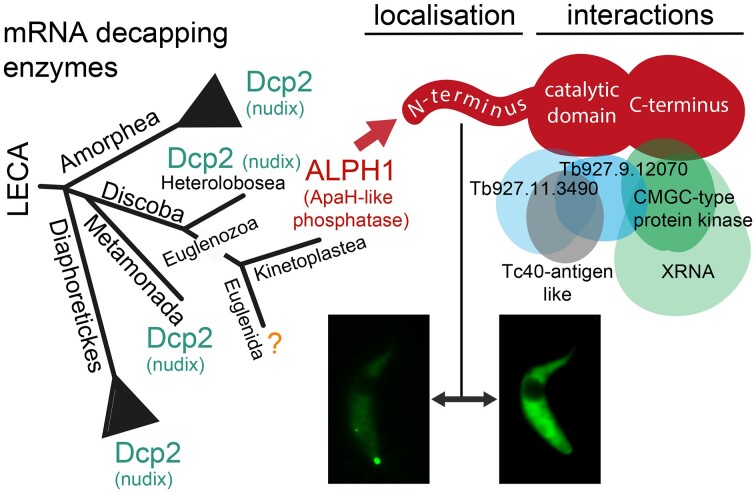

## INTRODUCTION

Eukaryotic 5′-3′ mRNA decay is initiated by removal of the poly(A) tail by a deadenylation complex, followed by 5′-end decapping and 5′-3′ exoribonucleolytic degradation. It is the dominant mRNA decay pathway in many eukaryotes, including yeast and Kinetoplastida, and is highly conserved.

The Kinetoplastida separated early from the main eukaryotic lineage but retain a conserved deadenylation complex (CAF1/NOT (1,2)) and conserved 5′-3′ exoribonuclease (XRNA (3,4)). However, the decapping reaction is mechanistically distinct as Kinetoplastida lack orthologues of the canonical decapping enzyme, the nudix domain protein Dcp2 and all associated factors (Dcp1, Edc1-3 and Pat1) that are present in other eukaryotes. Instead, the ApaH-like phosphatase ALPH1 (Tb927.6.640; UniProt ID: Q583T9) is the decapping enzyme in *T. brucei* (5) and likely all Kinetoplastida (6). A fraction of ALPH1 co-localizes with XRNA to a granular structure at the posterior pole of the cell (the PP-granule) that is devoid of most other RNA metabolizing proteins (5) and has no known function. ApaH-like phosphatases are unrelated to nudix-domain proteins but originate from the bacterial ApaH protein, a subgroup of the family of phosphoprotein phosphatases (PPP) (7,8).

Enzymes of the PPP family are present in all major branches of the eukaryotic lineage, albeit absent from some taxa, as for example mammals and land plants (6–8). Removal of an mRNA cap by ApaH-like phosphatases is novel in eukaryotes, and recent bioinformatics analysis indicates probably restricted to Kinetoplastida (6). There is evidence for a bacterial origin for this mechanism as some bacterial mRNAs are capped with a nucleoside-tetraphosphate cap under certain conditions, and these caps are removed by ApaH, suggesting that Kinetoplastida continue to use a prokaryote-derived mechanism (9–11).

Eukaryotic ApaH-like phosphatases remain poorly studied. To the best of our knowledge, apart from *T. brucei* ALPH1, only the Ppn2 ApaH-like phosphatase of *S. cerevisiae* had been experimentally characterized and is an endopolyphosphatase located within the vacuolar lumen (12). The substrate range of ApaH/ApaH-like phosphatases appears rather broad. Specifically, the bacterial ancestor enzyme ApaH cleaves pyrophosphate bonds of NpnN nucleotides where n ≥ 3 (13–15) and mRNAs capped with NpnN (9,11) and also possesses phosphatase and ATPase activity (16). Further, we recently demonstrated that three randomly chosen eukaryotic ALPH enzymes from three different eukaryotic lineages all possess mRNA decapping activity *in vitro*, even though all have predicted/demonstrated mitochondrial localizations rendering it unlikely that cytoplasmic mRNAs are physiological substrates (6). Despite this, Kinetoplastida ALPH1 has a highly specific role in mRNA decapping (5).

The Kinetoplastida decapping ALPH architectures are distinct from other eukaryotic ALPH proteins by possessing unique C-terminal extensions of ∼250 amino acids, and most also possess a unique N-terminal extension between ∼200 and 500 amino acids. These extensions contain no identifiable motifs or sequence similarities to other proteins, but are implicated in mediating regulation and substrate specificity (5,6). In contrast, the vast majority of eukaryotic ApaH-like phosphatases, like their bacterial ancestor, consist of just the catalytic domain (6).

Here, we define the composition of the *T. brucei* ALPH1 multi-protein decapping complex, investigate the functions of the unique C- and N- terminal extensions of ALPH1 and provide a model for function and regulation of the complex. The C- terminus of ALPH1 is more important for ALPH1 function, protein interaction and localization than the N-terminus, which is dispensable in *in vitro* culture. Proximity labelling identified a cohort of ALPH1 interacting proteins: With the exception of XRNA all are unique to Kinetoplastida and some interactions require either the N- or C- terminal domain of ALPH1. Confirmation by reverse isolations and localization studies, together with demonstration of conservation of complex composition in *Trypanosoma cruzi*, suggests a trypanosome decapping complex with intriguingly unique composition and hence mechanism.

## MATERIAL AND METHODS

### Structural analysis

Secondary structure and disorder predictions were done using PredictProtein (17). A predicted structural model created with the AlphaFold Monomer v2.0 pipeline (18) was downloaded from the AlphaFold DB version 27 January 2022 (19). Molecular graphics and analyses were performed with UCSF ChimeraX (20).

### Protein expression and purification

Full length ALPH1 (amino acid 1–734), ALPH1ΔN (amino acids 222–734), ALPH1ΔC* (amino acids 120–552) and ALPH1 catalytic domain (amino acids 222–552) were cloned into the pET28a-SUMO vector for transformation of BL21-CodonPlus(DE3)-RIL cells. Bacteria were cultured in LB medium. After reaching OD600 of 0.6–0.8 the expression of ALPH1 proteins was induced with 0.5 mM isopropyl-D-1-thiogalactopyranoside solution (IPTG) and conducted overnight at 25°C with 200 rpm shaking. After harvesting by centrifugation (4000 g, 4°C, 20 min) the bacteria pellet was resuspended in lysis buffer (30 ml per litre bacteria culture) (50 mM Tris pH 7.5, 0.5 M NaCl, 20 mM imidazole, 10% glycerol, 0.5 mM Tris(2-carboxyethyl)phosphine hydrochloride (TCEP), 1 mM PMSF, 0.5 μg/ml DNaseI and 35 mg lysozyme). The bacteria were lysed by sonication and the cell debris was removed by centrifugation at 48,880g for 30 min. From the supernatant, the ALPH1 proteins were first purified on a HisTrap HP column (GE Healthcare) equilibrated with lysis buffer lacking PMSF, DNaseI and lysozyme and the column was washed 4 times with the same buffer. The proteins were eluted with a 20 mM to 500 mM imidazole gradient (dissolved in 50 mM Tris pH 7.5, 0.5 M NaCl, 10% glycerol, 0.5 mM TCEP). All fractions that contained target proteins were combined, and diluted 1:5 in dilution buffer (50 mM Tris pH 8.5 (WT, cat) or pH 8.0 (ΔC*) or pH 7.5 (ΔN), 10% glycerol, 0.5 mM TCEP) to decrease salt concentration.

Proteins were loaded to HiTrap Q HP columns (WT, ΔN) or HiTrap Heparin HP columns (cat, ΔC*) equilibrated with buffer A (50 mM Tris pH as dilution buffer, 100 mM NaCl, 10% glycerol, 0.5 mM TCEP). The protein was eluted with a gradient of 100 mM to 500 mM NaCl in the same buffer. Protein-containing fractions were pooled and diluted 1:1 with dilution buffer at pH 7.5 and the His-SUMO tag was removed by overnight incubation with 200 μg self-made SUMO protease at 4°C. The last stage of protein purification was size exclusion chromatography performed on a Superdex 200 Increase 10/300 GL (GE Healthcare) (WT, ΔN) or Superdex 75 10/300 GL (GE Healthcare) (ΔC*, cat) pre-equilibrated in 50 mM HEPES pH 7.4, 150 mM NaCl, 5% glycerol, 0.5 mM TCEP. After purification, all proteins were flash frozen in liquid nitrogen and kept at -80°C.

### Size-exclusion chromatography – multiangle light scattering (SEC-MALS)

Size exclusion chromatography coupled with multiangle light scattering (MALS) was used for protein molecular mass determination. The experiment was performed using NGC Scout 10 Medium-Pressure Liquid Chromatography System (BioRad) connected to a light detector miniDAWN TREOS (Wyatt Technologies) and RefractoMax 520 Refractive Index Detector (ERC Inc). The ALPH1 full length/ΔN/ΔC*/cat (100 μl) were injected at 5 mg/ml on a Superdex 200 Increase 10/300 GL column (GE Healthcare) and run at 15°C with 0.4 ml/min flow rate in PBS buffer. The data were analysed using Astra 7 software.

### Trypanosomes and cell culture

*Trypanosoma brucei* Lister 427 procyclic cells were used for most experiments. All overexpression and RNAi experiments were done in Lister 427 pSPR2.1 cells that express a TET repressor (21); expression is induced with tetracyclin (1 μg/ml for 24 h). Cells were cultured in SDM-79 (22) at 27°C and 5% CO_2_. Transgenic trypanosomes were generated by standard procedures (23), using the Amaxa Nucleofector (Lonza Cologne AG, Germany) with home-made transfection buffer (24). All experiments used logarithmically growing trypanosomes. For starvation, one volume of cells was washed once in one volume PBS and cultured for 2 h in one volume PBS. Heat shock was done for 2 h at 41°C either in a thermoblock or waterbath. Actinomycin D was used at 10 μg/ml from a 1:1000 stock in DMSO, sinefungin at 2 μg/ml from a 1:1000 stock in water and cycloheximide at 50 μg/ml from a 1:100 stock in water. Monomorphic bloodstream forms *T. brucei* strain Lister 427 expressing VSG221 were cultivated at 37°C in 5% CO_2_ in HMI-9 containing 10% (v/v) fetal bovine serum; transfections were done as in procyclic cells.

*Trypanosoma cruzi* Lister Dm28c (25) epimastigote cells were used for all experiments. Cells were cultured at 28°C in liver infusion tryptose (LIT) medium supplemented with 10% heat-inactivated bovine fetal serum. All experiments used logarithmically growing trypanosomes of 3 days of culture. Transgenic trypanosomes were generated by transfection using Amaxa Nucleofector (Lonza Cologne AG, Germany) as previously described (26).

### Plasmids and cloning

For endogenous tagging of proteins with different tags we used either the tagging system from (27) or the PCR-based approach from (28). For inducible overexpression we used a T7-polymerase independent system based on the PARP promoter regulated by a TET operator (21). A full list with details on plasmids / PCR products used for the generation of all transgenic cell lines with tagged proteins described in this work is available as Supplementary Table S4. To delete one ALPH1 allele, a blasticidin resistance cassette was flanked by the 560 nts upstream of the ALPH1 ORF and 607 nts downstream of the ALPH1 ORF to allow deletion of ALPH1 by homologous recombination ( = SK390). For replacing one endogenous allele with ALPH1ΔN we cloned the 550 nts upstream of the ALPH1 ORF, a neomycin resistance cassette, the β-α tubulin intergenic region and the nts corresponding to amino acids 222–478 of ALPH1 ( = SK464) into a pJET cloning vector; the entire cassette was released with restriction enzymes at both sites and used for transfection. The control for the Turbo-ID experiment was an eYFP-TurboID-HA fusion protein that was inducibly expressed (21) ( = SK547). The ALPH1 RNAi plasmid was previously described (5).

For *T. cruzi* episomal TcXRNA-GFP tagged expression, the PCR product was cloned into the pDONR221^TM^ vector from Gateway Technology (Invitrogen) and was then recombined into a pTcGWGFP_N_NH vector (29). Transfections were performed as described above and cells were selected with geneticin (50μg/ml).

### Preparation of trypanosomes for imaging /HaloTag®

Trypanosomes expressing fluorescent proteins were fixed over-night in 2.8% paraformaldehyde (PFA) in PBS and washed in PBS prior to imaging. To visualize the CMGC-kinase fused to HaloTag, life cells were washed once in SDM-79, incubated with 1 μM HaloTag® TMR Ligand (Promega) in SDM-79 for 15 min, washed twice in PBS, fixed in 4% PFA for 20 minutes at RT, washed once with PBS/500 mM glycine, washed once with PBS and imaged. Cells were mixed 1:1 with DAPI (4′,6-diamidino-2-phenylindole, 5 μg/ml) on the slide prior to imaging.

### Microscopy

Z-stack images (100 stacks a 100 nm distance) were taken with a custom build TILL Photonics iMic microscope equipped with a sensicam camera (PCO AG, 6.45 μm/pixel) und 100x oil immersion (NA1.4) objectives (Olympus) (100 stacks a 100 nm distance). Filter sets were (i) ex: 320–380 nm, dc: 400–430 nm, em: 438–486 nm (DAPI), (ii) ex: 430–474 nm, dc: 585 nm, em: 489–531 nm (Alexa Fluor™488) and (iii) ex: 540–580 nm, dc: 585 nm, em: 592–664 nm (mChFP, Cy3). (iv) ex: 422–455 nm, dc: 450 nm, em1: 461–504 nm, em2: 525–561 nm (eYFP). Images were deconvolved using Huygens Essential software (SVI, Hilversum, The Netherlands) and are either presented as Z-stack projection of 100 images taken in 100 nm distances (sum slices). To quantify the fraction of a protein at the posterior pole, fluorescence was measured within a circle at the posterior pole (radius 12 pixels = 0.774 μm), background-corrected, and correlated with the background-corrected total fluorescence of the cell.

The HaloTag® cells (Figure 6B untreated and heat shock) were imaged with the inverted wide-field microscope Leica DMI6000B (Leica Mi-crosystems GmbH, Wetzlar, Germany) equipped with a100x oil immersion (NA 1.4) and a Leica DFC365 camera (6.5 μm/pixel). Filter sets were (i) ex: 340–380 nm, dc: 400nm, em: 450–490 nm (DAPI), (ii) ex: 450–490 nm, dc: 495nm, em: 500–550 nm (eYFP), (iii) ex: 530–560nm, dc: 570 nm, em: 572- 648 nm (TMR). Images are presented as Z-stack projection (sum slices) of 5 slices, without deconvolution.

### FISH experiments combined with immunofluorescence

Fluorescence *in situ* hybridization and immunofluorescence was done as previously described (30), except that we used oligos with a direct fluorophore (Cy3 on both ends). The sequence used to probe for the poly(A) tail was T_30_, the sequence to probe the miniexon was: CAATATAGTACAGAAACTGTTCTAATAATAGCGTT. The experiment was controlled by the respective sense oligos.

### Northern blots

Northern blots were done as previously described (31). rRNA and total mRNA probing was done using oligos antisense to the 18S rRNA (5′-CCTTCGCTGTAGTTCGTCTTGGTGCGGTCTAAGAATTTC-3′) or antisense to the miniexon sequence of the spliced leader RNA (5′-CAATATAGTACAGAAACTGTTCTAATAATAGCGTT-3′), coupled to IRDye800 and IRDye700, respectively.

### Western blots and antibodies

Western blots were performed using standard procedures. Biotinylated proteins were detected with IRDye800 CW streptavidin and the HA tag was detected with rat-anti-HA (3F10, Sigma). ALPH1 antiserum was raised against recombinant ALPHΔN protein fused to an N-terminal 6xHis tag, that was expressed in RosettaTM (DE3) competent *E. coli* (Novagen). ALPH1ΔN was extracted from the soluble fraction of the bacterial cell lysate via nickel affinity using standard procedures and used to immunize rabbits. Serum was affinity-purified against recombinant ALPH1ΔN protein and specificity was tested on both Western blot and immunofluorescence, using an ALPH1 RNAi cell line as well as transgenic ALPH1 cell lines as controls (Supplementary Figure S2). Sadly, the antibody lost activity after 2 years and is not available any longer.

### Cryomill affinity capture of protein complexes from *T. brucei*

XRNA was expressed as C-terminal mNeonGreen fusion and ALPH1 as N- or C-terminal eYFP fusion, respectively, all from the endogenous locus in procyclic *T. brucei* cells. Cells were harvested, subjected to cryomilling and affinity capture, essentially as described previously, except that for XRNA-mNeonGreen mNeonGreenTrap magnetic agarose (Chromotek) was used (32,33). In brief, 2 litre cultures of PCF trypanosomes at a density of approximately 8 × 10^6^ cells/ml were harvested at 1500*g and washed once with serum free SDM-79. Resuspended cells were then sedimented by centrifugation (1500*g) into a capped 20 ml syringe placed in a 50 ml Falcon tube. After discarding the supernatant, inserting the plunger and removing the cap the cells were passed slowly into liquid nitrogen in order to form small pellets suitable for subsequent cryomilling. These cell pellets were processed by cryomilling at 77 K into a fine powder in a modified planetary ball mill (Retsch) (34). Six smidgen spoons of cell powder were suspended in 6 ml ice-cold buffer A (20 mM HEPES pH7.4, 250 mM NaCl, 0.5% CHAPS, complete EDTA-free protease inhibitor cocktail (Roche)), sonicated with a microtip sonicator (Misonix Ultrasonic Processor XL) at setting 4 (∼20 W output) for 5 × 1 second, transferred into six Eppendorf LoBind tubes and insoluble material was removed by centrifugation (20000 g, 10 min, 4°C). The clear lysate was incubated with either 3 μl mNeonGreen Trap magnetic agarose (Chromotek), or GFP Trap magnetic agarose (Chromotek) for 30 min on a rotator, then washed three times with buffer A. After pooling the 6 samples, captured protein was eluted by incubation in 30 μl 4 x NuPAGE LDS sample buffer (ThermoFisher), supplemented with 2 mM dithiothreitol, at 72°C for 15 minutes and then run 1.5 cm into a NuPAGE Bis-Tris 4–12% gradient polyacrylamide gel (ThermoFisher). The respective gel region was sliced out and subjected to tryptic digest and reductive alkylation using standard procedures. Eluted proteins were analysed by LC-MSMS on an Ultimate3000 nano rapid separation LC system (Dionex) coupled to an Orbitrap Fusion mass or Q-exactive mass spectrometer (Thermo Fisher Scientific). At least 3 replicate experiments were performed. Wild type cells (at least 3 replicates) served as control.

### Affinity capture of protein complexes from *T. cruzi*

Cytoplasmic lysates from *T. cruzi* Dm28c expressing TcXRNA-GFP or TcALPH1-GFP were generated after cell disruption through cavitation as described (35) with modifications. Logarithmically growing cells were harvested by centrifugation at 3.000g at RT, washed once with ice cold PBS, followed by resuspension in lysis buffer (20 mM HEPES-KOH, pH 7.4, 75 mM potassium acetate, 4 mM magnesium acetate, 2 mM DTT, supplemented with cOmplete™ Protease Inhibitor Cocktail, from Merck) to a concentration of 1 × 10^9^ cells/ml. The resuspended cells were transferred into the Cell Disruption Vessel 4639 (Parr) and incubated at 4°C under 70 bar pressure for 40 min, followed by rapid decompression. The lysates were microscopically controlled for completion of cell lysis and then centrifuged at 17,000g for 10 min to remove cellular debris.

The immunoprecipitation assays were performed using anti-GFP nanobodies (36) bound to Dynabeads® M-270 Epoxy magnetic beads, as previously described (34). 1 ml lysate was incubated with 3 ul anti-GFP magnetic beads at 4°C under agitation for 1–2 h. The beads were washed three times with lysis buffer and proteins were eluted by boiling in sample buffer for 5 minutes. For mass spectrometry, the eluted proteins were loaded onto 13% SDS-PAGE gels and allowed to migrate into the resolving gel. Gel slices containing the whole IP products were then excised and submitted to an in-gel tryptic digestion and mass spectrometry analysis as previously described (37,38).

### Affinity enrichment of biotinylated proteins and on-beads tryptic digests

The expression of TurboID fusion proteins was induced for 24 h with tetracyclin, unless expression was from the endogenous locus. No extra-biotin was added, as we found the biotin concentration in the SDM79 medium (827 nM) to be sufficient for high levels of biotinylation. 5 × 10^8^ cells were harvested at a cell density of 1 × 10^6^ to 10^7^ cells per ml at 1400 *g*, washed once with serum-free medium and pellets were rapidly frozen in liquid nitrogen and stored at -80°C. For isolation of biotinylated proteins, each cell pellet was resuspended in 1 ml lysis buffer (0.5% octylphenoxypolyethoxyethanol (IGEPAL), 0.1 M piperazine-N,N′-bis(2-ethanesulfonic acid) (PIPES)-NaOH pH 6.9, 2 mM ethylene glycol-bis(β-aminoethyl ether)-N,N,N′,N′-tetraacetic acid, 1 mM MgSO_4_, 0.1 mM ethylenediaminetetraacetic acid (EDTA), complete protease inhibitor cocktail (Roche)) and incubated for 15 min at room temperature in an orbital mixer. Soluble and non-soluble fractions were separated by centrifugation (14 000 *g*, 5 min, 4°C) and the soluble fraction incubated with 100 μl streptavidin-linked Dynabeads (MyOne Streptavidin C1, Thermofisher) for 1 h at 4°C under gentle mixing. Beads were washed twice in 1 ml buffer 1 (2% (w/v) SDS in water) once in 1 ml buffer 2 (0.1% (w/v) deoxycholate, 1% Triton X-100, 1 mM EDTA, 50 mM HEPES pH7.5, 500 mM NaCl), once in 1ml buffer 3 (250 mM LiCl, 0.5% IGEPAL, 0.5% (w/v) deoxycholate, 1 mM EDTA, 10 mM Tris-HCl pH 8.1) and once in 1 ml buffer 4 (50 mM Tris-HCl pH 7.4, 50 mM NaCl); each washing step was eight minutes at room temperature (RT) under orbital shaking. Beads were then prepared for tryptic digestion by washing three times in 500 μl ice-cold 50 mM NH_4_HCO_3_, resuspension in 40 μl of the same buffer supplemented with 10 mM dithiothreitol and incubation in a thermomixer at RT for 1h. Iodoacetamide was added to a concentration of 20 mM, followed by incubation in the dark at RT for 30 min. Finally, 5 μg/ml proteomics-grade trypsin (SOLu-Trypsin, SigmaAldrich) was added to the beads. The digest was done overnight at 30°C in a thermomixer (1000 rpm). After removal of the first eluate, beads were resuspended in 50 μl 50 mM NH_4_HCO_3_ supplemented with 10 mM dithiothreitol and 5 μg/ml mass spectrometry (MS) grade trypsin and incubated in a thermomixer at 37°C for 1h. The eluate was combined with the first eluate, and both were lyophilized in a Speed-vac (Christ alpha 2–4). Peptides were resuspended in 50 mM NH_4_HCO_3_ and passed over C_18_ stage tip columns as described (39). After removal of polymers by the HiPPR procedure (Thermo Fisher Scientific), peptides were analysed by liquid chromatography-tandem mass spectrometry (LC-MSMS) on an Ultimate3000 nano rapid separation LC system (Dionex) coupled to an LTQ Q-exactive mass spectrometer (Thermo Fisher Scientific). LoBind tubes (Eppendorf) were used throughout. Triplicate experiments were performed for each cell line.

### Analysis of proteomics data

Spectra were processed using the intensity-based label-free quantification (LFQ) in MaxQuant version 1.6.16 (40,41). LFQ data were analysed using Perseus (42). For statistical analysis, LFQ values were log_2_ transformed and missing values imputed from a normal distribution of intensities around the detection limit of the mass spectrometer. These values were subjected to a Student's *t*-test comparing an untagged control (*wt* parental cells) triplicate sample group to the bait triplicate sample groups.

For BioID, a second control, cells expressing a eYFP-TurboID fusion, served to identify proteins biotinylated in a non-specific manner. -log_10_*t*-test *p*-values were plotted versus *t*-test difference to generate multiple volcano plots (Hawaii plots). Potential interactors were classified according to their position in the Hawaii plot, applying cut-off curves for significant class A (SigA; FDR = 0.01, s0 = 0.1), significant class B (SigB; FDR = 0.05, s0 = 0.1) and significant class C (SigC; FDR = 0.05, s0 = 2.0). The cut-off is based on the false discovery rate (FDR) and the artificial factor s0, controlling the relative importance of the *t*-test *p*-value and difference between means (At s0 = 0 only the *p*-value matters, while at non-zero s0 the difference of means contributes).

For XRNA affinity capture and for the CMGC-kinase BioID, enrichment ratios were calculated in addition to the statistical analysis.

### Phylogenetic analyses

ALPH1 and the five core interactors (determined in this study), that are all localizing to the PP granule, were subjected to sequence analysis in HMMER (43). For ALPH1, Tb927.9.12070 and the CMGC family kinase Tb927.10.10870 only partial sequences that discriminate from paralogs with different function, were used: For ALPH1 the C-terminal region (residues 528–773) was used to discriminate from the paralog ALPH2, which has non-cytoplasmic localization in *T. brucei* and likely functions outside mRNA metabolism (6,44). For the CMGC family kinase the serine threonine kinase domain (Supplementary Figure S7A) was excluded (using the C-terminal region, residues 392–570). For Tb927.9.12070 (Supplementary Figure S7B), the N terminal region (residues 1–276) was used to exclude the similarity region with the RNA helicase Tb927.4.3890 (Supplementary Figure S7C). For XRNA Tb927.7.4900 (Supplementary Figure S7D), the C-terminal region (residues 585–1418) was used to exclude 5′-3′ exoribonuclease paralogs, like XRN2 that functions in transcription termination (45). *E. gracilis* XRNA homologs were not found in the HMMER search but detected in the transcriptome (46). The distribution of Dcp2 and its prototypic interactors was analysed in parallel using Dcp2 (P53550), Dcp1 (Q12517), Edc1 (P53080), Edc2 (P40023), PNRC2 (Q9NPJ4), Edc3 (P39998) and Pat1/PatL1 (P25644/Q86TB9) sequences from *S. cerevisiae* and *H. sapiens*. For Edc4 (Q6P2E9) a partial sequence (residues 463–1043) was used to exclude the widespread N-terminal WD40 repeat domain.

## RESULTS

### Modelling predicts the ALPH1 N-terminal extension as unstructured and the C-terminal extension as α -helical

**Figure 1.**
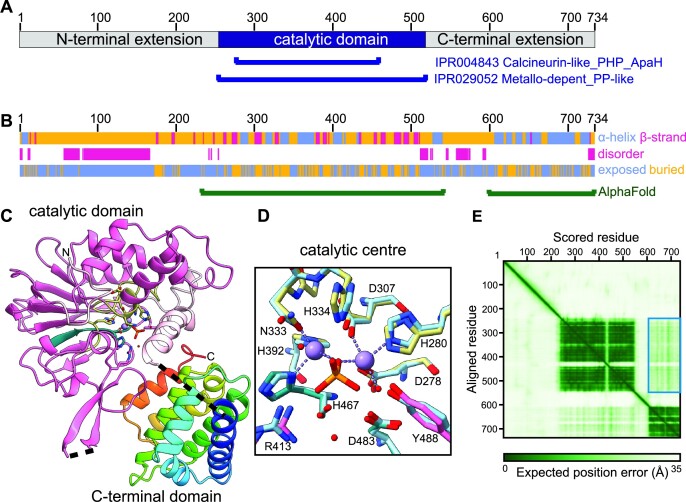
**Predicted structure of ALPH1. (A)** Schematics of ALPH1: the protein consists of a catalytic domain with N- and C- terminal extensions. (**B**) Secondary structure predictions for ALPH1. The N-terminal region of ALPH1 is predicted to be mostly unstructured, exposed and disordered, with the longest almost continuous disordered region at residues 57–166. The central region responsible for the catalytic activity contains a predicted α/β-domain which overlaps with the annotated Metallo-dependent phosphatase-like region (Interpro superfamily IPR029052, residues 253–519). The catalytic domain is followed by a disordered linker and a small C-terminal domain comprised of α-helices. (**C-E**) Structural model of ALPH1 predicted by AlphaFold. Shown in cartoon depiction are the catalytic domain (residues 253–543, in pink) and the C-terminal domain (residues 598–734, in rainbow). Regions with low model confidence (the N-terminal region and two disordered linkers at residues 421–437 and 544–597) were omitted. The central part of the catalytic domain overlaps well with ALPH2 crystal structure (PDB entry 2QJC, (47)) with 44% sequence identity in this region (residues 263–511, in hot pink). Highlighted are key residues forming the active site coming from the 4 phosphatase motifs (in khaki) and 2 ALPH motifs (in teal) (6). The inset (**D**) shows superposition of these residues with those from ALPH2 (PDB entry 2QJC, in light blue), including two Mn^2+^ ions (purple), 3 water molecules (their oxygens in red) and a phosphate ion (orange). (**E**) The predicted aligned error for the AlphaFold model (available at the AlphaFold Protein Structure Database entry for UniProt ID Q583T9) indicates two domains with little confidence regarding their mutual orientation (values above 15Å in the region corresponding to the inter-domain accuracy for the catalytic and C-terminal domains, indicated by the blue rectangle).

*T. brucei* ALPH1 comprises 734 amino acids and consists of a catalytic domain, flanked by N-terminal and C-terminal extensions of similar size (Figure 1A). We analysed the sequence and predicted structure of ALPH1, which suggests that the N-terminal region is largely unstructured, whereas the C-terminal region contains a novel, structured domain connected to the central catalytic domain by a disordered linker (Figure 1B). An AlphaFold model of ALPH1 is in agreement with these observations (Figure 1C). The N-terminal extension, up to residue 250, is predicted as a continuous disordered region with per-residue confidence score (pLDDT) <50 (omitted in Figure 1C) suggesting that this region is unstructured (18). The catalytic domain is predicted to closely resemble the crystal structure of *T. brucei* ALPH2 (PDB entry 2QJC (47)) followed by a linker containing an additional α-helix (residues 528–542) that docks in the vicinity of the active site and possibly regulates accessibility. The active site residues of the trypanosome ALPH1 model superimpose with the *T. brucei* ALPH2 active site (Figure 1D), including those residues coordinating divalent cations (6). The C-terminal region (598–743) was modelled as a small, structured domain comprised of seven α-helices. A PDBeFold (48) search with the latter domain model revealed structural similarity to the Ge-1 domain of *Drosophila melanogaster* Edc4 (pdbID 2VXG; 105 residue structural alignment; Q-score = 0.28) (49). Edc4 (enhancer of decapping 4) is a metazoan specific decapping factor that bridges the Dcp1-Dcp2 interaction while interacting with XRN1 (50,51). Whereas the position of the α-helix (528–542) at the C-terminus of the catalytic domain is predicted confidently, the relative orientation of the catalytic and C-terminal domains is uncertain as indicated by predicted aligned error values in excess of 15Å (Figure 1E). The linker connecting these two domains was predicted with low pLDDT score and is likely mostly unstructured. Although it cannot be excluded that the C-terminal domain interacts with and partially occludes the catalytic domain regulating accessibility, we conclude that the mutual positioning of these domains in the AlphaFold model is likely largely artificial, and we have no evidence suggesting their interaction.

### ALPH1 is a dimer and dimerization requires the C-terminus

**Figure 2.**
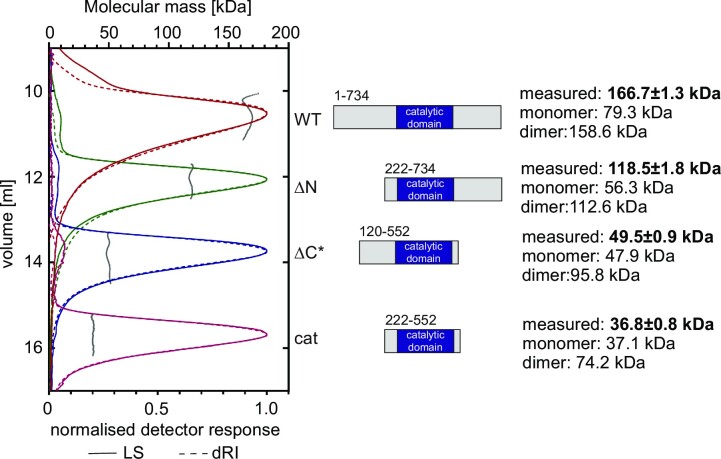
ALPH1 is a dimer in solution. Size exclusion chromatography coupled with multiangle light scattering was used for protein molecular mass determination of wild type ALPH1 (WT), ALPH1ΔN (amino acids 222–734), ALPH1ΔC* (amino acids 120–552) and ALPH1cat (amino acids 222–552). All proteins were expressed in *E. coli* fused to a His-SUMO tag, purified using Nickel-affinity chromatography followed by Ion-exchange chromatography (Q or Heparin column) and the His-SUMO tag was cleaved off with SUMO protease. All proteins were loaded at a concentration of 5 mg/ml. Full length ALPH1 and ALPH1ΔN elute as a dimer, while ALPH1ΔC* and ALPH1cat elute as monomers.

We produced recombinant ALPH1 to characterize its oligomeric state. ALPH1 was expressed in *E. coli* fused to an N-terminal His-SUMO Tag and purified using nickel-affinity chromatography. The tag was removed with a SUMO protease and the molecular mass of the recombinant protein analysed by size exclusion chromatography coupled with multiangle light scattering (SEC-MALS) (Figure 2). ALPH1 has a predicted molecular weight of 79.3 kDa but elutes with a molecular weight of 166.7 ± 1.3 kDa, indicating it is dimeric. The experiment was repeated with ALPH1 lacking the N-terminal domain (ALPH1ΔN; amino acids 222–734, theoretical molecular weight: 56.7 kDa) with similar results: ALPH1ΔN elutes at 118.5 ± 1.8 kDa and is thus also dimeric. ALPH1 truncations lacking the C-terminal domain, either with most of the N-terminus still intact (ALPH1ΔC*, amino acids 120–552, theoretical molecular weight 47.9 kDa) or without the N-terminus (ALPH1cat; amino acids 222–552, theoretical molecular weight 36.7 kDa) elute at 49.9 ± 0.5 and 36.8 ± 0.8 kDa respectively, indicating that both proteins are monomeric. Hence ALPH1 is dimeric in solution and dimerization requires the C-terminus.

### The N-terminus of ALPH1 mediates localization to the posterior pole but is dispensable in cultured trypanosomes or for targeting to stress granules

**Figure 3.**
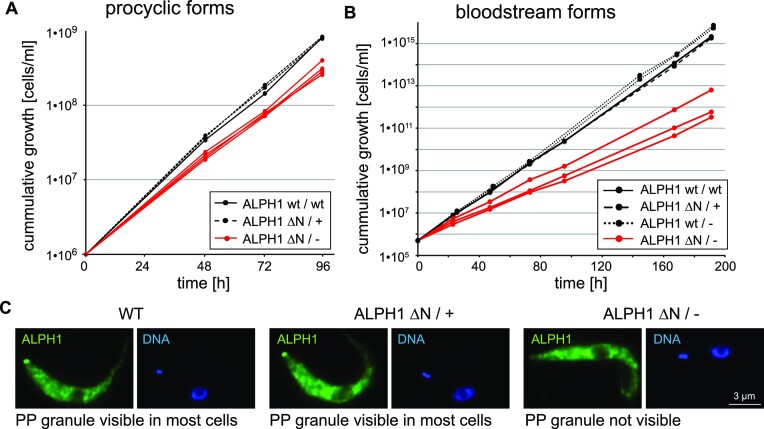
ALPHΔN/– cells are viable. (**A**) Cumulative growth of procyclic cells of WT (one clone), ALPHΔN/+ (2 clones) and ALPHΔN/– (4 clones). (**B**) Cumulative growth of bloodstream form cells of WT (one clone), ALPHΔN/+ (one clone), ALPH1 WT/– (2 clones) and ALPHΔN/– (3 clones). (**C**) ALPH1 was detected in WT, ALPHΔN/+ and ALPHΔN/– cells by immunofluorescence with ALPH1 antiserum. One representative images is shown for each cell line.

The N-terminus of ALPH1 is poorly conserved between Kinetoplastida (5) and entirely absent in the ALPH1 homologues of *Trypanosoma grayi* and *Leptomonas pyrrhocoris* (6). It is predicted as unstructured (Figure 1) and it is dispensable for dimerization (Figure 2). All the data point towards a non-essential function of this domain. To test this possibility, one allele of ALPH1 was replaced by an N-terminal truncated ALPH1 (ALPH1ΔN; 222–734) to create ALPH1ΔN/+, and the second allele replaced by a blasticidin resistance gene to create ALPH1ΔN/–. Correct insertions were confirmed by western blotting (Supplementary Figure S1). Generation of clones of ALPHΔN/– cells was facile in two different trypanosome life cycle stages (bloodstream and procyclic forms) with only a minor proliferative defect (Figure 3A and B). We conclude that the N-terminus of ALPH1 is nonessential in culture, albeit the impact to proliferation indicates a decreased fitness.

**Figure 4.**
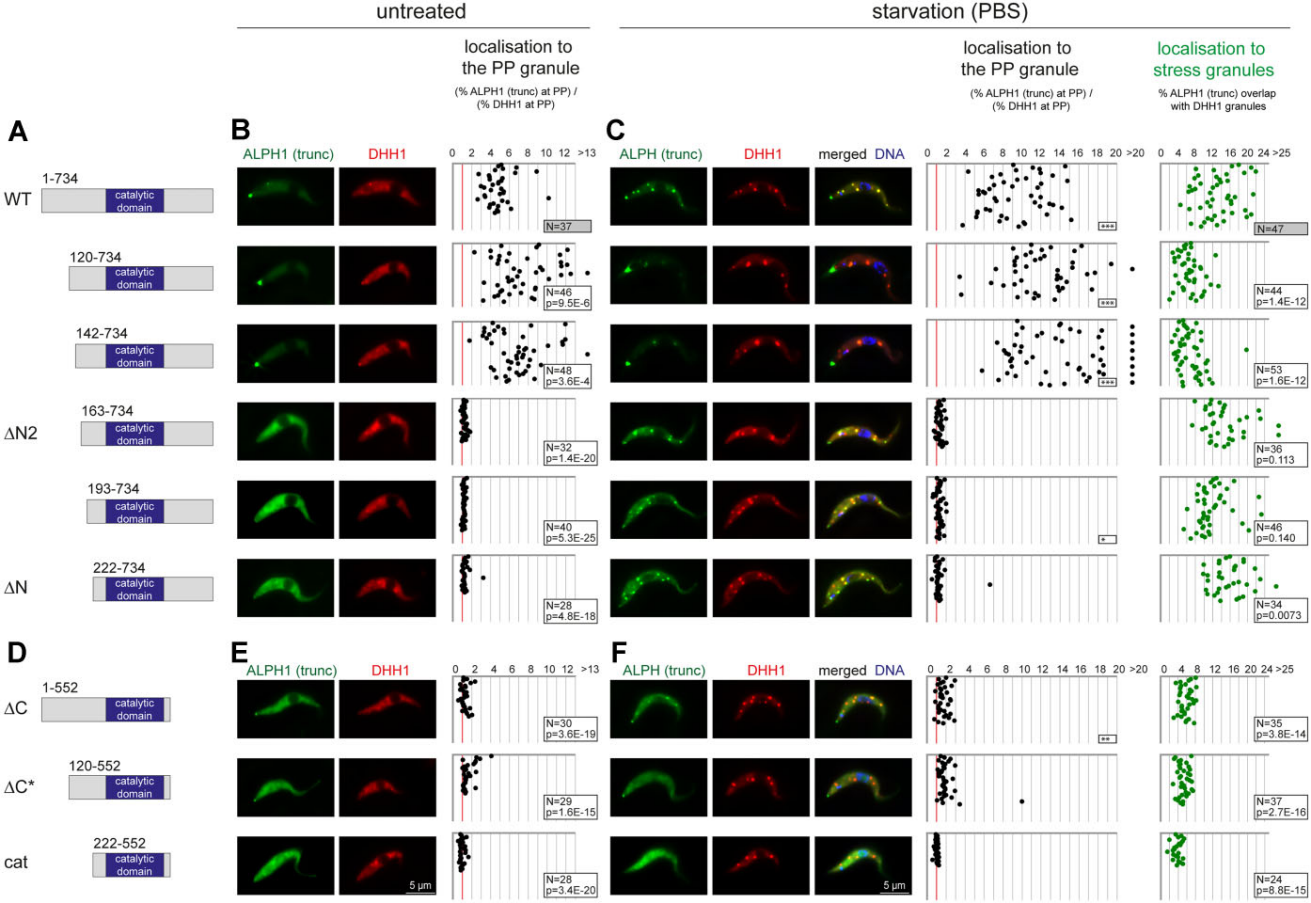
Localization of ALPH1 truncations. (**A, D**) Overview about ALPH1 truncations used in this study. Numbers refer to amino acid positions. Wild type ALPH1 and all truncations were expressed with an inducible expression system in cells expressing mChFP-DHH1 from the endogenous locus. (**B, E**) Left: Representative microscopy images of untreated cells. Right: Posterior pole localization of ALPH1 (truncations) was quantified by dividing the percentage of ALPH1 at the PP granule through the percentage of DHH1 in the same region. Any value above 1 (red line) indicates localization to the posterior pole. Each dot represents one cell. The number of cells is indicated in the figure (N). A two-tailed unpaired homoscedastic TTEST (p) was used to test the significance of any decrease or increase in PP localization in comparison to WT ALPH1. (**C, F**) Left: representative microscopy images of starved cells. DHH1 served as a stress granule marker. Middle: Posterior pole localization of ALPH1 (truncations) was quantified as above. A significant increase in PP localization in comparison to untreated cells is marked with ***, ** or * correlating to the result of a two-tailed unpaired homoscedastic TTEST of <0.0005, <0.005 or <0.05, respectively. Right: The percentage of ALPH1 (truncations) in stress granules was quantified. Granules were identified by the granule Marker DHH1. Each dot represents one cell. The number of analysed cells is indicated in the figure (N). A two-tailed unpaired homoscedastic TTEST (p) was used to test the significance of any decrease or increase in stress granule localization in comparison to WT ALPH1.

A hallmark of ALPH1 and XRNA is localization to the posterior pole (5,31). Interestingly, immunofluorescence of ALPH1ΔN/– cells with ALPH1 antiserum (Supplementary Figure S2) indicated a complete loss of ALPH1 localization from the posterior pole (Figure 3C). To investigate further, we expressed ALPH1-eYFP and N-terminally truncated versions via inducible overexpression (21) in cells also expressing mChFP-DHH1, which does not localize to the PP-granule, from the endogenous locus (Figures 4A and B). This resulted in an approximately 4-fold increase in the level of ALPH1-eYFP within 24 h of induction but with no significant effects on ALPH1 localization, proliferation or global mRNA levels over 96 h (Supplementary Figure S3).

We quantified ALPH1 PP localization by dividing the percentage of ALPH1 within a defined circular area at the posterior pole by the percentage of DHH1 fluorescence in the same area (Figure 4B). Any value above one indicates ALPH1 localization to the PP granule, as DHH1 does not localize to the PP. An N-terminal truncation of up to 141 amino acids showed no reduction in ALPH1 PP localization, but rather a significant increase from an average of 4.8 (WT ALPH) to 7.4 (*P* = 9.5E-6) and 6.9 (*P* = 0.0004) for ALPH120-734 and ALPH142-737, respectively. Additionally removing 21 or more amino acids from the ALPH1 N-terminus leads to near complete loss of PP granule localization, indicating that posterior pole targeting requires amino acids 142 to 162 of the ALPH1 N-terminus.

Most RNA binding proteins, including *T. brucei* ALPH1 and DHH1, localize to stress granules on starvation (5). Stress granules are aggregates of proteins and RNAs and for a protein to localize requires interactions with RNA and/or RNA binding proteins. We quantified stress granule localization of ALPH1 and ALPH1 N-terminal truncations, using mChFP-DHH1 as a stress granule marker (Figure 4C). The general tendency in PP localization efficiency was unaffected by starvation, albeit there was a significant increase in PP localization (*P* << 0.0005) for the ALPH1 variants with efficient PP localization (WT, 120–734 and 142 734), indicating that starvation stress triggers PP localization. By contrast, when the fraction of ALPH1 in stress granules was quantified, we found that stress granule localization of most N-terminal ALPH1 truncations was similar to the *wild type* protein. The only exceptions were ALPH1120-734 and ALPH1142-734 that had significantly reduced (*P* << 0.0005) stress granule localization, likely caused by their increased localization to the PP granule.

In conclusion, while the N-terminal domain of ALPH1 is required for efficient localization to the posterior pole, it is neither required for proliferation in culture nor for localization to starvation stress granules, suggesting that it does not contain elements essential for function.

### The C-terminus is required for efficient localization of ALPH1 to RNA granules

The C-terminus of ALPH1 is more conserved among Kinetoplastida than the N-terminus, is predicted α-helical and required for ALPH1 dimerization (Figures 1 and 2). C-terminally truncated ALPH1 failed to efficiently localize to the posterior pole, similar to ALPH1 truncated at the N-terminus by 162 amino acids or more (Figure 4D and E). In contrast to N-terminally ALPH1 truncations, stress granule localization of ALPH1ΔC was also significantly reduced (*P* << 0.0005) (Figure 4F), indicating loss of interaction with RNA and/or RNA binding proteins. Additional removal of the N-terminus caused a complete loss in both posterior pole localization and stress granule localization (Figures 4D–F). The C-terminus is thus required for ALPH1 localization to any type of granule, suggesting a major role in mediating ALPH1 interactions.

### ALPH1 interacts with multiple partners

Dcp2, the canonical eukaryotic mRNA decapping enzyme, is part of a multisubunit complex. The N- and C- terminal regions of Dcp2 engage in multiple protein-protein interactions essential for recruitment of mRNAs and adoption of an active conformation (52). To investigate whether ALPH1 is similarly part of a larger complex, we examined the ALPH1 interactome by proximity labelling, fusing ALPH1 to TurboID biotin ligase by endogenous tagging. To gain domain resolution of interaction sites we additionally carried out TurboID experiments with truncated versions of ALPH1 (ΔN2, ΔC, cat, compare Figure 4) which required overexpression. As a control we included TurboID analysis of overexpressed full-length ALPH1. All proximity labelling experiments were controlled by parental, i.e. untagged, cells. In addition, cells expressing an eYFP-TurboID fusion, served to identify proteins biotinylated by the TurboID enzyme in a non-specific manner. All fusion proteins also contained an HA-epitope tag.

**Figure 5.**
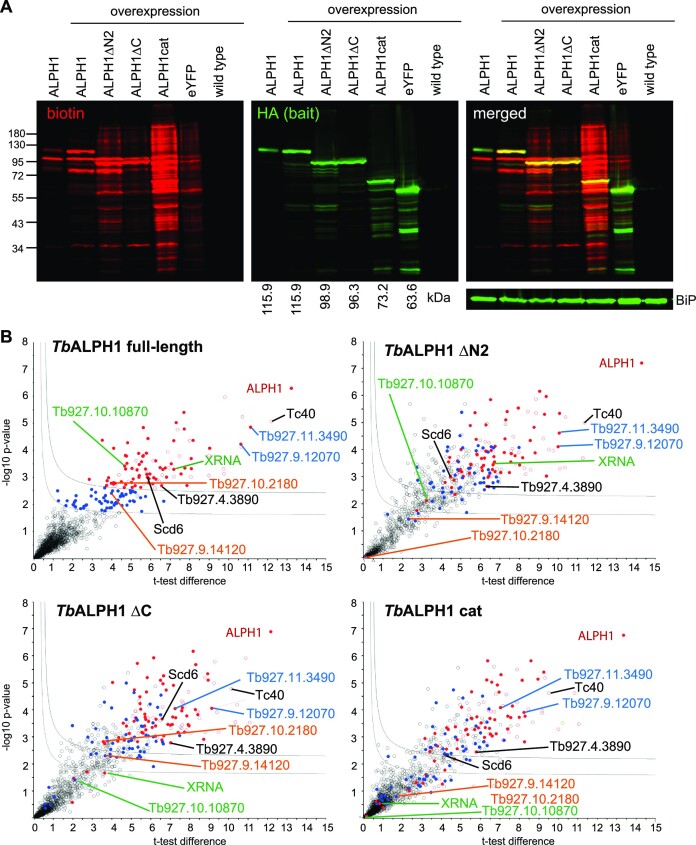
BioID proximity labelling with ALPH1. (**A**) Western blot loaded with cell extracts from cells expressing TurboID-HA fusions of ALPH1 (endogenous expression and inducible overexpression) and truncated ALPH1 (inducible overexpression). Wild type cells and cells expressing eYFP-TurboID-HA served as controls. The blot was probed with IRDye 800CW streptavidin to detect biotinylated proteins (shown in red) and with rat-anti-HA (3F10, Sigma) followed by IRDye 680RD goat anti-rat IgG to detect the bait proteins (shown in green). (**B**) Hawaii-plot (multiple comparative volcano plots) for statistical analysis of BioID experiments with full-length ALPH1 and the three truncated fragments ΔN2 (lacking the N-terminal domain), ΔC (lacking the C-terminal domain) and the catalytic fragment (cat; lacking both, the N- and C-terminal domain). All samples were prepared in triplicates. To generate the volcano plots, the − log_10_*P*-value was plotted versus the *t*-test difference (difference between means), comparing each respective bait experiment to the *wt* control. Potential interactors were classified according to their position in the plot, applying cut-off curves for ‘significant class A’ (SigA; FDR = 0.01, s0 = 0.1) and ‘significant class B’ (SigB; FDR = 0.05, s0 = 0.1). Detected protein groups are coloured for their respective significance class in the full-length experiment (red = SigA; blue = SigB). Selected proteins are labelled (green for apparent C-terminal interactors; orange for apparent N-terminal interactors; blue for interactors with posterior pole localization, that do not rely on the presence of the terminal regions). Three further proteins that may be part of the decapping complex based on experiments with other baits (see below) are shown in black. For all data see Supplementary Table S1; a comparison to a BioID experiment with endogenously expressed ALPH1, and a respective correlation analysis, is shown in Supplementary Figure S4.

All generated cell lines were verified by Western blotting using streptavidin to detect biotinylated proteins and anti-HA to detect the bait (Figure 5A). Biotinylated proteins were readily detectable in all lines except for parental control. For all fusion proteins, the bait protein was among the most abundant biotinylated proteins. Cells expressing full length ALPH1 from either the endogenous locus or via overexpression had a similar pattern of biotinylated proteins: the apparent band for the 116 kDa ALPH1 fusion protein was more dominant in the over-expresser line, and, as expected, absent from cells expressing truncated ALPH1 versions. Cells expressing ALPHΔN2 and ALPHΔC exhibited distinct patterns of biotinylated proteins. In contrast, cells expressing the catalytic domain of ALPH1 alone had distinctly higher intensity and more extensive labelling. The eYFP-TurboID control showed considerable biotinylation, despite the absence of specific interactions, and was used in the following as a stringent control to define potential bystander labelling (53).

Next, all cell lines were subjected to streptavidin affinity purification followed by LC-MSMS analysis. Proteins enriched by BioID, compared to controls, were grouped into confidence intervals (SigA, SigB), based on statistical analysis in Perseus (42) (Supplementary Table S1A). To further eliminate false positives from low level bystander labelling, all quantified protein groups were filtered to require at least three quantification values within all 18 samples (Supplementary Table S1B).

Endogenously tagged ALPH1-TurboID yielded 70 significantly enriched proteins (16 in SigA, 54 in SigB). Overexpressed ALPH1-TurboID yielded 150 significantly enriched proteins (95 in Sig A (red in Figure 5B), 55 in SigB (blue in Figure 5B). BioID with the ALPH1 overexpression thus identified more proteins, but with correlation between proteins identified in both experiments: 67 of 70 significantly enriched proteins from the endogenous experiments were likewise significantly enriched with the overexpression and 75 out of the remaining 83 proteins that were unique to the overexpression experiment were also enriched in the endogenous experiment, albeit outside of SigA/B. In fact, the 150 proteins falling into SigA/B of the overexpression experiment correlate with a Pearson coefficient of 0.90, decreased to 0.79 when considering all 1571 detected proteins (Supplementary Figure S4). BioID experiments with truncated versions of ALPH1 resulted in the identification of 285/160, 214/116 and 194/69 proteins significantly enriched in SigA/SigB in comparison to parental cells for ΔN2, ΔC and cat respectively (Supplementary Table S1B); most were undetected with the eYFP control. The larger number of ALPH1cat candidate interactors compared to full-length ALPH1 is likely a result of a less defined localization of ALPH1cat (Figure 4).

### A high-confidence list of ALPH1 interacting partners

**Figure 6.**
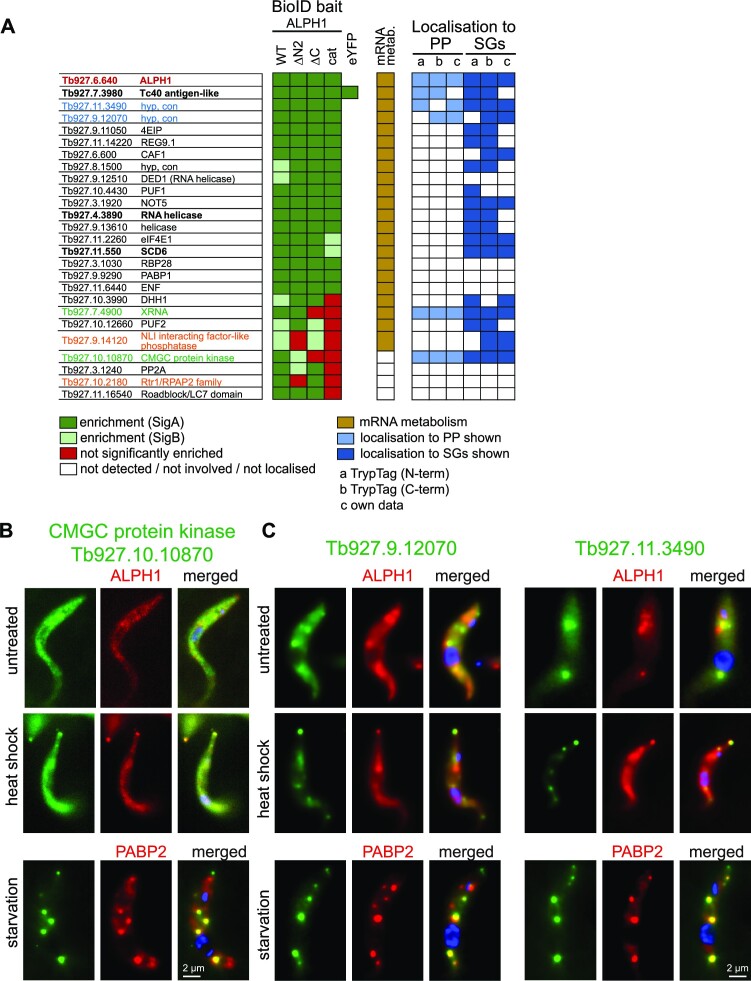
Analysis of the ALPH1 interactome. (**A**) The final high-confidence table of potential ALPH1 interacting proteins identified by BioID (Supplementary Table S1D). The color-scheme indicates whether a protein was identified with the respective ALPH1 BioID bait and in which significance group (green/red), whether the protein is involved in mRNA metabolism (light brown), and whether the protein is known to localize to either the posterior pole (PP, light blue) or to starvation stress granules (SGs, dark blue) as judged by Tryptag (44) or own data (published here or elsewhere). None of the proteins was detected or enriched with the eYFP control, with the exception of the Tc40-antigen like protein. (**B**) Tb927.10.10870 was expressed as a HaloTag® fusion and stained with TMR in a cell line also expressing ALPH1-eYFP from the endogenous locus. Representative images of one untreated and one heat shocked cell (2 h 41°) are shown (Z-stack projection sum slices of 5 slices a 140 nm); note that colors are switched for clarity. Tb927.10.10870 fused to eYFP was also co-expressed with PABP2-mCHFP (a marker for starvation stress granules (31)) and starvation was induced by incubation in PBS for 2 h. Images are presented as deconvolved Z-stack projections (sum slices). (**C**) Tb927.9.12070 and Tb927.11.3490 were expressed fused to a C-terminal eYFP tag from the endogenous locus in a cell line also expressing ALPH1-mChFP or the stress granule marker protein PABP2-mChFP from the endogenous locus. Fluorescent images of representative cells are shown under untreated conditions, after 2 h heat shock at 41°C and after 2 h of PBS starvation as projections (sum slices) of deconvolved Z-stacks. Note that the autofluorescence of the lysosome is visible in the red channel, because the ALPH1-mCHFP fluorescence is very weak.

For constructing a high-confidence list of candidate interacting proteins for ALPH1 and its truncations we included only proteins (i) present in both BioID experiments with the full length ALPH1 (endogenous and overexpression) with at least SigB (67 protein groups, Supplementary Table S1C), (ii) absent from both SigA and SigB of the TurboID-eYFP control (47 of these 67 proteins), and (iii) with either a clear connection to mRNA metabolism, (experimentally proven or possessing a classical RNA binding domains (21 of these 47 proteins)) or absent from at least one of the ALPH1 truncations, suggesting a specific interaction with a domain (a further five of these 47 proteins). Additionally we retained Tb927.7.3980 (Tc40-antigen like), as, despite detection with the TurboID-eYFP control (SigA) it has both posterior pole localization (44) and a connection to mRNA metabolism (44,54,55) and hence is potentially a genuine interactor. Moreover, we excluded a WD domain G-beta repeat protein Tb927.4.960, as this protein is identified in many BioID experiments with unrelated proteins (e.g. Mex67 and NUP158; (53) and is enriched in the eYFP control (albeit below the significance interval), indicating a likely common BioID contaminant. The final list contains 26 proteins, including ALPH1 (Figure 6A and Supplementary Table S1D).

### ALPH1 BioID hits with localization to the posterior pole

ALPH1 is distinct from most other trypanosome RNA-binding proteins by its localization to the posterior pole. Among the high confidence ALPH1 BioID hits were five proteins with a posterior pole localization (44): XRNA, a CMGC-family protein kinase Tb927.10.10870, hypothetical proteins Tb927.11.3490 and Tb927.9.12070 and the Tc40 antigen-like protein.

XRNA has a known co-localization with ALPH1 to the posterior pole (5,31) and its function in the 5′-3′decay pathway is well established (3,4)). Interestingly, the ALPH1 BioID approach identified XRNA as a significant interactor, but exclusively with full length ALPH1 or ALPH1ΔN. For ALPH1cat and ALPH1ΔC, XRNA enrichment is below the SigB threshold, at 10% for ALPH1ΔC and at 6% for ALPH1cat compared to full length ALPH1 (Figure 5B and 6A). These data indicate an interaction between ALPH1 and XRNA dependent on the C-terminus of ALPH1.

Tb927.10.10870 encodes a CMGC-family protein kinase with no known connection to mRNA metabolism. Endogenous expression of a HALO-tag fusion protein of the kinase in a cell line expressing ALPH1-eYFP from the endogenous locus showed co-localization of both proteins to the posterior pole, most prominent after heat shock (Figure 6B). At starvation, an eYFP-fusion of the kinase co-localized with PABP2 to starvation stress granules, in addition to the posterior pole granule, a strong indication of a function in mRNA metabolism (Figure 6B). The domain interaction pattern of the CMGC kinase with ALPH1 resembles that of XRNA, i.e. significantly enriched with ALPH1ΔN as bait, but dramatically reduced with ALPH1ΔC and absent with ALPH1cat (Figure 5B). In contrast to XRNA, the kinase is also less enriched with ALPH1ΔN (SigB instead of SigA), suggesting that both ALPH1 termini are important for the interaction, but with a more significant C-terminal contribution.

The 87 kDa protein encoded by Tb927.11.3490 has no predicted domains, while the 109.0 kDa protein product of Tb927.9.12070 has a C-terminal predicted ATP-dependent RNA helicase domain. Both proteins co-purify with oligo(dT) beads, indicating mRNA association (55). We expressed these proteins fused to a C-terminal eYFP-tag and confirmed heat-shock inducible relocalization to the posterior pole with ALPH1 and co-localization with PABP2 to starvation stress granules (Figure 6C). Both proteins are enriched in BioID isolations with all ALPH1 truncations, indicating that they likely bind the ALPH1 catalytic domain.

Tc40-antigen-like protein is the *T. brucei* ortholog to the Tc40 antigen from the related parasite *T. cruzi*, an immunodominant antigen in chronic Chagas disease patients (56). In *T. brucei*, many lines of evidence support a function for this protein in mRNA metabolism: Tc40 is enriched in purified starvation stress granules (57), localizes to nuclear periphery granules, a special RNA granule type of trypanosomes that forms at the cytoplasmic site of nuclear pores when trans-splicing is inhibited (54) and is captured by oligo(dT) beads (55).

### ALPH1 interactors specific to defined ALPH1 regions and domains

Most proteins identified with the BioID experiments are enriched with all ALPH1 truncations, including the isolated catalytic domain, but eight proteins, including XRNA and the CMGC-family kinase (discussed above), are absent or significantly less enriched with the ALPH1 catalytic domain when compared to full-length protein, indicating domain specific interactions (Figure 6A). Of this cohort, two proteins are also absent from potential ALPH1ΔN interactions, indicating an interaction requiring the N-terminal domain: the Rtr1/RPAP2 family protein (Tb927.10.2180) and a nuclear LIM interactor (NLI)-interacting factor-like phosphatase (Tb927.9.14120). Rtr1/RPAP2 has not been identified by any mRNA-related screen and localizes to the cytoplasm with both N- and C- terminal tagging (44). In contrast, the NLI interacting factor-like phosphatase contains a CCCH motif (58), has been identified as a post-transcriptional repressor (55,59), localizes to a trypanosome-specific RNA granule type at the nuclear periphery (54) and thus is implicated in mRNA metabolism. The protein localizes to granules (44) and we have expressed the protein as a C-terminal eYFP fusion together with the stress granule marker PABP2 fused to mChFP and confirmed that these granules are stress granules starvation-induced stress granules (Supplementary Figure S5A).

Of the four proteins potentially interacting with both the C- and N-terminal region of ALPH1, but not the catalytic domain, two have clear connections to mRNA metabolism: the pumilio protein PUF2 is essential (60), coprecipitates with oligo(dT) and acts as a co-transcriptional repressor (55). The DEAD box RNA helicase DHH1 plays a role in life-cycle-dependent mRNA regulation (61). The remaining two proteins appear unrelated to mRNA metabolism: The serine threonine phosphatase 2A (Tb927.3.1240) has not been localized by TrypTag and, in our C-terminal endogenous tagging, we saw no evidence for localization to the posterior pole granule or starvation stress granules (Supplementary Figure S5B and S5C). The Roadblock/LC7 domain containing protein Tb927.11.16540 has axonemal localization (44) and thus a potential function in flagellar transport. We conclude that these latter two proteins are likely mis-identifications and not components of the decapping complex.

### The trypanosome mRNA decapping complex

Two ALPH1 BioID interactors were selected for confirmation: the CMGC-type protein kinase and XRNA. XRNA functions downstream of ALPH1, colocalizes with ALPH1 to the posterior pole, and the yeast ortholog XRN1 is a known Dcp2 interactor (62,63): an interaction is therefore likely. The CMGC-type protein kinase resembles XRNA in its localization to the posterior pole and confined interaction to the ALPH1 C-terminal domain; its function is unknown.

**Figure 7.**
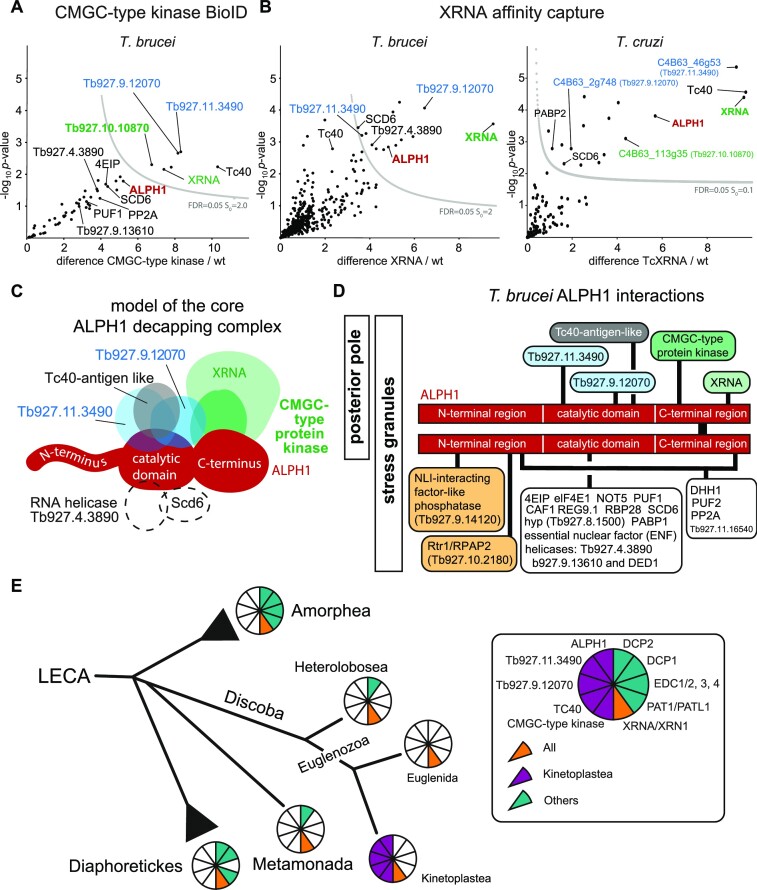
The trypanosome decapping complex. (**A**) Volcano plot of the CMGC-type kinase interactome. The CMGC-type protein kinase Tb927.10.10870 was expressed as TurboID fusion and biotinylated proteins were purified by streptavidin affinity and identified by LC-MSMS. Shown is a volcano plot generated by plotting the − log_10_*P*-value versus the *t*-test difference, comparing the bait experiment (TurboID- tagged Tb927.10.10870) to a wild type (wt) control. All proteins of the final ALPH1 BioID list are labelled. The statistical cut-off (SigC; FDR = 0.05 S0 = 2.0) is indicated by a gray curved line. The full dataset is shown in Supplementary Table S2. (**B**) Volcano plot of the XRNA interactome in *T. brucei* and *T. cruzi*.XRNA was extracted from trypanosome cells via GFP (*T. cruzi*) or mNeonGreen (*T. brucei*) nanobody beads and co-purified proteins were identified by LC-MSMS. Shown are volcano plots for XRNA affinity capture in *T. brucei* (left) and *T. cruzi* (right). Plots were generated by plotting the −log_10_*P*-value versus the *t*-test difference, comparing the bait experiment (XRNA) to a wild type (wt) control. All proteins that are also in the final stringent list of the ALPH1 BioID are labelled. The statistical cut-off (FDR = 0.05 S0 = 2) is indicated by a grey line. The full datasets are shown in Supplementary Table S3. (**C**) A model of the ALPH1 core complex. Overlapping proteins from interactors of ALPH1, CMGC-type protein kinase and XRNA are shown as the most likeliest components of the ALPH1 core complex. Proteins with slightly less robust evidence for being complex subunits are shown in dashed circles. Note that the dimerization of ALPH1 via the C-terminus observed *in vitro* is not shown here for clarity. Moreover, we cannot exclude that binding sites of either ALPH1 domain are used in competition rather than simultaneously. (**D**) Summary of the ALPH1 interactome. ALPH1 is depicted as a schematic homodimer and all interacting proteins identified in this work are shown connected to the respective ALPH1 domain(s). Localization of proteins to the posterior pole and/or to stress granules is indicated at the left. (**E**) Evolution of distinct components in decapping complexes in Kinetoplastida. Coulson plot representation of subunit presence layered onto a simplified eukaryotic phylogeny, to emphasize subunit losses and replacements of subunits between Metamonads and Discoba. In teal are canonical subunits including Dcp1/2 and Edc, while in magenta are the subunits reported here as associated with APLH1. Significantly, XRNA/XRN1 (orange) is retained by all lineages. The selective pressure and order of events, such as by a gradual or more catastrophic change its unknown, but parallels several additional systems such as the lamina and kinetochore (78,79). Significantly, in Metamonada, Heterolobosea and Euglenida subunit retention is sparse and may reflect the presence of an additional set of divergent components remaining to be identified. For a list of gene IDs for decapping complex component homologs see Supplementary Table S5.

The CMGC kinase interactome was determined with BioID, expressing the protein from its endogenous locus as a C-terminal TurboID-HA fusion. Biotinylated proteins were readily detectable on a Western blot probed with streptavidin (Supplementary Figure S6) and mass spectrometry identified 216 protein groups (Supplementary Table S2A). Of these, 54 protein groups were enriched in comparison to the control BioID (parental cells) and identified in at least two of three replicates (Figure 7A; Supplementary Table S2B). From these 54 proteins we excluded all proteins also identified with the eYFP control in SigA or B, with the exception of the Tc40 antigen-like protein (see above). From the remaining 44 proteins, we excluded all proteins that were less than 2.5-fold enriched. The final list of putative CMGC-type kinase interactors contains 22 proteins (Supplementary Table S2C). Only five proteins of this list fall into the SigC group (FDR = 0.05, s0 = 2.0): next to the bait protein this is XRNA, the two ALPH1 catalytic domain interactors Tb927.11.3490 and Tb927.9.12070 and the Tc40-antigen-like protein. The other 17 proteins fall below the SigC significance interval, but include seven additional proteins in the final ALPH1 BioID list, including ALPH1. The lower significance of the latter protein cohort can be explained by low abundance of the protein kinase.

To investigate the XRNA interactome, we used immunoprecipitation. XRNA was expressed fused to a C-terminal mNeonGreen tag from its endogenous locus (31). Cells were broken by cryo-milling and the XRNA complex captured by mNeonGreen affinity immunoisolation. Mass spectrometry detected 1484 protein groups in the eluate (Supplementary Table S3A), out of which 223 were enriched in comparison to parental cells and had at least two quantification values within the four bait samples (Supplementary Table S3B). Of these, 14 were considered significantly enriched (FDR = 0.05, s0 = 2.0; Supplementary Table S2C), including the bait. This cohort of 13 high-confidence XRNA interactors included five proteins that were also in the ALPH1 BioID list: ALPH1 itself, SCD6, RNA helicase Tb927.4.3890 and the two RNA binding proteins Tb927.11.3490 and Tb927.9.12070 (Figure 7B, left). Notably, the CMGC-kinase was not identified: one possible explanation is a transient or less-stable interaction. The Tc40 antigen like protein was enriched, but below the chosen significance threshold.

In parallel, to demonstrate conservation of the decapping complex architecture among trypanosomatids, we chose *Trypanosoma cruzi*, a prominent parasitic pathogen. We targeted XRNA by affinity immunoisolation and largely recapitulated the *T. brucei* interactome (Figure 7B, right, Supplementary Table S3). *T. cruzi* XRNA immunoprecipitated ALPH1 together with orthologues of Tb927.11.3490 (C4B63_46g53) and Tb927.9.12070 (C4B63_2g748). In contrast to the affinity capture in *T. brucei*, both the CMGC-type kinase C4B63_113g35 (Tb927.10.10870 in *T. brucei*) and the Tc40 antigen-like protein (C4B63_52g40) were significantly enriched. Some stress-granule proteins were also enriched, but a significant difference was absence of the RNA helicase C4B63_44g238 (Tb927.4.3890 in *T. brucei*), identified in both the *T. brucei* ALPH1 BioID and XRNA affinity capture.

Altogether, these data, provide compelling evidence for an mRNA decapping complex that contains ALPH1, XRNA and the CMGC-family kinase. Of 22 putative CMGC-family kinase interactors, 12 were also identified for ALPH1 and five for *T. brucei* XRNA. Of the 13 *T. brucei* XRNA interactors, five were in both the ALPH1 and CMGC-kinase BioID isolations. To define a robust core of the putative *T. brucei* mRNA decapping complex, we included only proteins identified with all three bait proteins. A so-defined mRNA decapping complex consist of six proteins, all with posterior pole localization: ALPH1, XRNA, the CMGC-family kinase, the RNA binding proteins Tb927.11.3490 and Tb927.9.12070 and the Tc40-antigen-like protein (coloured in Figure 7C). Two further proteins are potential complex components, but with less robust support (dashed circles in Figure 7C): The RNA helicase Tb927.4.3890 and SCD6 do not localize to the posterior pole and are below significance in the CMGC-kinase BioID. The ortholog of the helicase is also absent from the *T. cruzi* XRNA pulldown. A summary of all ALPH1 interactions discovered in this work is shown in Figure 7D.

### Direct immunoprecipitation of ALPH1 delivers a potential sub-complex

While we confirmed the BioID derived composition of the ALPH1 decapping complex by reverse experiments targeting CMGC-type kinase and XRNA, we also attempted a direct affinity capture of ALPH1 from *T. brucei* and *T. cruzi*, under the same respective conditions applied for XRNA. For C-terminal eYFP fusion, an epitope tag that is well tolerated in an ALPH1 knockout background (5), most interactors, including XRNA, were absent and only Tb927.11.3490 and the Tc40-antigen like protein were captured (Supplementary Figure S10A, B; Supplementary Table S6). Fusing eYFP N-terminally to TbALPH1 lead to similar results, delivering the latter two proteins and Tb927.9.12070, albeit at low enrichment (Supplementary Figure S10C; Supplementary Table S6). It is tempting to speculate that the ALPH1 fusion-tag is fully buried in the rather large decapping complex, thus inaccessible to the nanobody and, as a consequence, only a complex with partial composition precipitates. The presence of such a sub-complex, co-existing in a spatially (or temporally) distinct manner is conceivable and possibly biologically relevant.

### The ALPH1 mRNA decapping complex is Kinetoplastida specific

ALPH1 is conserved among kinetoplastids, with the exception of the free-living Bodonid *Bodo saltans* (6), that does however encode orthologues of Tb927.11.3490, Tb927.9.12070, the TC40 antigen-like protein and the CMGC-family kinase, indicating incomplete data as a potential reason for ALPH1 absence. Indeed, a tblastn search readily detected a genomic region (CYKH01001277 position 14 169–15 402) with a partial coding sequence homologous to *T. brucei* ALPH1 (R272-A551(E-value: 2e-58); G607-L662(E-value: 5.8)) that appears to be syntenic, as judged by the upstream presence of the evolutionary conserved gene encoding the RRP45 exosome subunit (BSAL_92565 and Tb927.6.670) (64). Orthologs of the core interactors with PP localization are present in most Kinetoplastida genomes (Figure 7E). Tb927.11.3490 and the TC40 antigen-like protein are not detected in any genome beyond the kinetoplastids and the other two interactors have unique sequence stretches: The N-terminal 390 residue serine/threonine kinase domain of the CMGC-family kinase is flanked by a C-terminal region of variable length within Kinetoplastida (Supplementary Figure S7A). Tb927.9.12070 shares partial similarity with the RNA-helicase Tb927.4.3890 (Supplementary Figure S7B, C), an ALPH1 interactor which does not localize to the PP, but conditionally, to stress-granules. This RNA-helicase appears to be universally distributed, while Tb927.9.12070, again, has Kinetoplastida specific regions (Supplementary Figure S7B). Interestingly, the shared sequence of approximately 520 residues between the two Kinetoplastida helicases includes the helicase C-terminal domain but excludes the helicase ATP-binding domain (Supplementary Figure S7C). XRNA and SCD6 are universally distributed (Supplementary Figure S7D) and are known Dcp2 interactors in animals and fungi (62).

Surprisingly, neither ALPH1 nor the four core interactors could be detected in Heterolobosea, Metamonada or Euglenids (only represented by the draft genome/transcriptome of *Euglena gracilis*)*. E. gracilis* encodes an ApaH like phosphatase, but it consists of the catalytic domain only and therefore likely is not functioning in mRNA decapping, but rather the orthologue to the non-cytoplasmic *T. brucei* ALPH2 or to ALPH3 (Londoño et al., 2021). Whilst the prototypic Dcp2 interactors, Dcp1, Pat1 and Edc1-4, are all absent in Discoba and Metamonada, a short Dcp2 variant is present in some Heterolobosea and Metamonada genomes (Figure 7E; Supplementary Figure S7E).

### The posterior pole is a highly dynamic structure anterior of the microtubules plus end that is not enriched in mRNAs

All five proteins of the ALPH1 core complex have behaviours that distinguish them from other RNA metabolism proteins by virtue of posterior pole localization, indicating a potential function of this structure in mRNA decapping. We therefore characterized this structure in greater detail.

**Figure 8.**
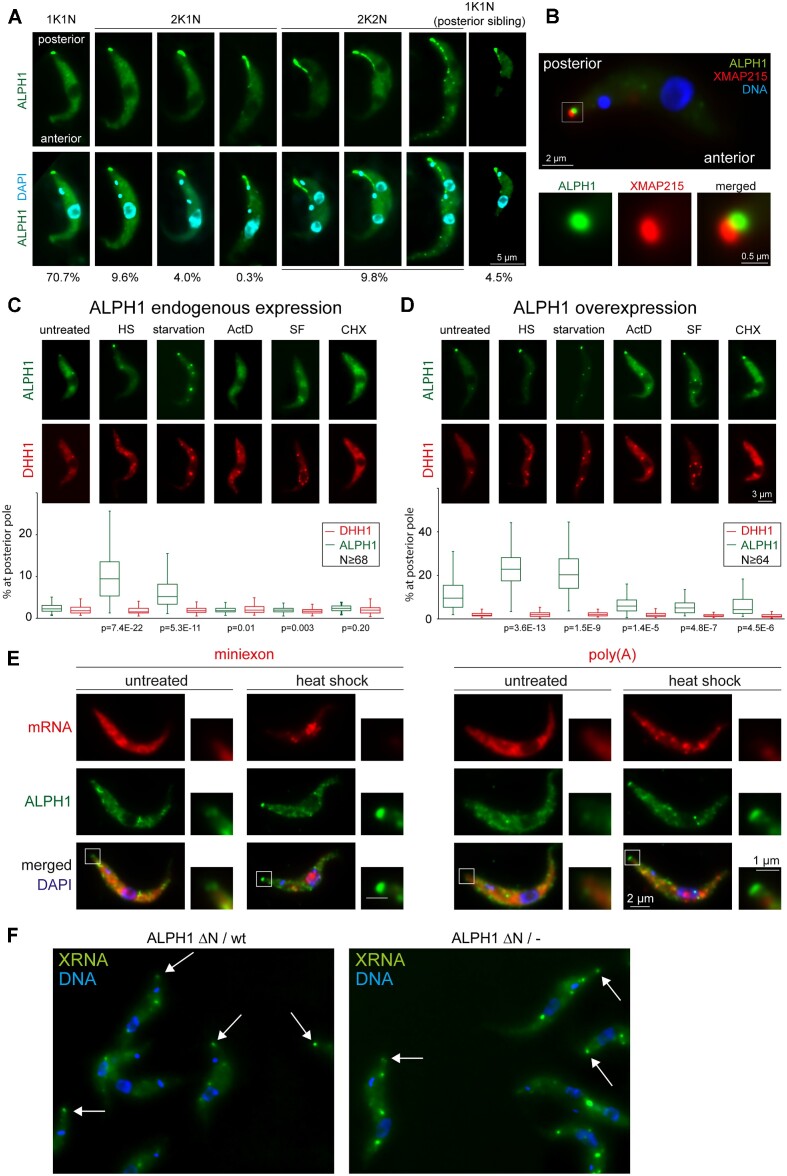
The posterior pole granule. (**A**) Changes in ALPH1 localization during the cell cycle. Cells were induced to overexpress ALPH1-eYFP for 24 h and representative images of the different cell cycle phases are shown. The different ALPH1 localizations were quantified from 376 cells and are shown as percentages underneath the images. (**B**) ALPH1 localizes posterior to XMAP215. Expression of ALPH-eYFP was induced by tetracyclin for 24 h in cells also expressing the microtubules plus end marker protein mChFP-XMAP215 from the endogenous locus. One representative cell is shown with the region of the posterior pole granule enlarged. (**C**, **D**) Changes in ALPH1 localization to the PP with mRNA-metabolism drugs. Cells co-expressing ALPH1-eYFP-4Ty1 and mChFP-DHH1 were left untreated or exposed for 1 h to 41°C (HS), starved for 2 h in PBS (starvation), incubated for 1 h with actinomycin D (ActD), sinefungin (SF) or cycloheximide (CHX). ALPH1 was either expressed from the endogenous locus (**C**) or overexpressed (24 h TET) (**D**). The percentage of ALPH1 at the posterior pole was quantified. The *P*-values are the result of an unpaired, two-tailed *t*-TEST and indicate the likelihood that the fraction of ALPH1 localization to the posterior pole is different between treated and untreated cells. (**E**) The PP-granule contains no mRNAs. Cells expressing ALPH1-4Ty1-eYFP were probed for total mRNA by oligos antisense to either the miniexon sequence (left) or the poly (A) tail (right). ALPH1 was detected by immunofluorescence using anti-Ty1 (BB2). Both untreated and heat-shocked cells were used; the later to increase the amount of ALPH1 at the posterior pole. Note that the miniexon probe also recognizes the nuclear-localized SL RNA next to total mRNAs; heat shock reduces total mRNA levels, but not SL RNA levels and thus causes an increase in the nuclear signal (31). (**F**) XRNA-eYFP was expressed from the endogenous locus in either wild type cells or ALPH1ΔN/– cells. XRNA localization was monitored after 70 minutes of heat shock. An arrow points to XRNA-eYFP localized at the posterior pole.

First, localization of overexpressed ALPH1-eYFP was monitored during the cell cycle (Figure 8A). The cell cycle stage of an individual cell can be determined from a DAPI stained image according to the number and position of kinetoplast(s) (the DNA containing structure within a single mitochondrion) and nuclei (65,66). Kinetoplast duplication takes place prior to nuclear division, resulting in three main cell cycle stages: 1K1N (one kinetoplast and one nucleus), 2K1N (two kinetoplasts and one nucleus) and 2K2N (two kinetoplasts and 2 nuclei). After division, the posterior nucleus moves between the two kinetoplasts and the longitudinally occurring cytokinesis produces a posterior and an anterior sibling. The majority of cultured cells are 1K1N cells and all have a spot-like, ALPH1-positive posterior pole granule. In 2K1N cells, we observed gradual elongation of the ALPH1 spot with the progressing cell cycle, indicated by an increasing distance between the two kinetoplasts. In very late 2K1N cells and all 2K2N cells, ALPH1 was visible as a punctate string starting at the posterior pole. In late 2K2N cells this string terminated proximal to the kinetoplast of the anterior sibling. After cell division, the string remains visible in a fraction of 1K1N cells that were most likely posterior siblings from a recent cell division, as they were of a small size and had a non-dividing kinetoplast.

This dynamic localization of ALPH1 in dividing cells is reminiscent of that previously described for the microtubule plus end marker protein XMAP215 (44,67), as the plus ends of the subpellicular microtubule array lie at the posterior end of the cell (68). We examined whether XMAP215 and ALPH1 co-localize, with XMAP215 expressed as an N-terminally tagged mChFP fusion protein from the endogenous locus in an inducible ALPH1 overexpression cell line. Both XMAP215 and ALPH1 localized to a spot at the posterior pole with ALPH1 slightly more anterior than XMAP215 (Figure 8B). In 63% of 1K1N cells (26/41) ALPH1 was unequivocally anterior to XMAP215 and in 33% in (14/41) cells ALPH1 appeared adjacent or co-localizes with XMAP215, and in only 2% of the cells (1/41) ALPH1 appeared to be slightly posterior to XMAP215 (Supplementary Figure S8A–C). Both XMAP215 and ALPH1 localize to a similar, but not entirely identical string-like structure in dividing cells (Supplementary Figure S8D). The data indicate that ALPH1 localizes to a structure that is associated but not identical with the microtubules plus ends, or perhaps to an anterior subcomplex of the structure.

The presence and size of most RNA granules depends on the state of mRNA metabolism. The percentage of ALPH1-eYFP at the posterior pole granule was quantified for both endogenously expressed and overexpressed ALPH1-eYFP in cells subjected to various treatments affecting mRNA metabolism (Figure 8C and D). As a control, each cell line also expressed the cytoplasmic RNA granule marker mChFP-DHH1 from the endogenous locus. When ALPH1-eYFP was expressed from the endogenous locus, on average, 2.6% of ALPH1-eYFP was at the posterior pole. This fraction significantly increased to 9.8% and 6.7% when cells were heat-shocked or starved, respectively. No significant changes were observed when cells were treated with the trans-splicing inhibitor sinefungin, the transcription inhibitor actinomycin D, or the translation inhibitor cycloheximide. When ALPH1-eYFP was overexpressed, an average of 11.6% of ALPH1-eYFP was at the posterior pole and this fraction increased to 22.9% and 21.1% when cells were heat-shocked or starved. Treatment with actinomycin D, sinefungin or cycloheximide caused a significant decrease in ALPH1 at the posterior pole to 6.6%, 5.7% and 6.3%, respectively. In both expression systems, the fraction of DHH1 at the posterior pole was unchanged. DHH1 was undetectable at the posterior pole granule, with the exception of occasional minor accumulation in ALPH1 overexpression cells. In conclusion, ALPH1 localization to the posterior pole increases with stresses that dissociate polysomes (heat shock, starvation) and thus increase free cytoplasmic mRNAs, but decreases with treatments that reduce free cytoplasmic mRNAs (sinefungin, actinomycin D, cycloheximide), suggesting a dynamic localization dependent on free mRNA concentration.

To determine if mRNA decapping occurs at the PP-granule, we asked whether the granule is enriched in mRNAs using fluorescence *in situ* hybridization (FISH) (Figure 8E). We probed untreated or heat-shocked cells with fluorescently labelled oligonucleotides, either antisense to the miniexon (present at the 5′end of trypanosome mRNAs) or antisense to the poly(A) tail, and monitored ALPH1 localization by immunofluorescence in parallel. While ALPH1 was clearly visible at the posterior pole, with the strongest signal in heat-shocked cells, there was no accumulation of mRNAs at the posterior pole visible detected with either FISH probe, even in heat-shocked cells (Figure 3D), consistent with a lack of mRNA decay intermediates at the posterior pole reported previously (69). Absence of both total mRNA and mRNA decay intermediates strongly argues against a function of the PP granule in mRNA degradation or storage.

Next, we asked whether ALPH1 localization to the posterior pole is required to recruit other members of the complex. We used the ALPH1ΔN/– cell line, which has no ALPH1 at the posterior pole (Figure 3C and 4), to test if the localization of XRNA at the posterior pole depends on ALPH1. XRNA-eYFP was expressed in wild type cells and in ALPH1ΔN/– cells from the endogenous locus and XRNA localization to the PP monitored by fluorescence microscopy in response to heat shock. XRNA remains present at the PP in ALPH1ΔN/– cells, indicating localization independent of ALPH1 (Figure 8F). Vice versa, we asked whether XRNA is required for ALPH1 localization to the posterior pole. We expressed a C-terminal HALO-tag fusion of ALPH1 in a previously characterized XRNA RNAi cell line (70). ALPH1 localization to the posterior pole was reduced within 24 h of RNAi induction, both in untreated and in heat-shocked cells (Supplementary Figure S9). These data suggest that XRNA localization to the posterior pole may be required to recruit ALPH1. An alternative explanation is that the reduction in ALPH1 localization to the posterior pole is caused by the change in mRNA metabolism that results from the RNAi depletion of XRNA.

In conclusion, the posterior pole is unlikely a place of mRNA decapping as it is neither enriched in mRNAs nor in mRNA metabolism products; this is consistent with findings that ALPH1 localization to the PP is nonessential for cellular survival. However, ALPH1 localization to the PP is highly dynamic and may serve to regulate overall decapping activity.

## DISCUSSION

Here, we define a decapping complex with a composition that appears unique to Kinetoplastida, suggesting a distinct mechanism and evolutionary origin underpinning this central process within this lineage (6). Whilst we find some similarities to the canonical Dcp2 decapping complex of animals and fungi, for example an interaction with the 5′–3′ exoribonuclease XRN1 and with SCD6, the core complex is largely composed of lineage-specific proteins and decapping relies on an ApaH-like phosphatase unique to Kinetoplastida. Moreover, there is great potential for exploiting this complex with central function in mRNA metabolism as a drug target against diseases caused by Kinetoplastida, as the entire ApaH like phosphatase family is absent from mammals (6–8).

### The ALPH1 N-terminal domain is dispensable, while the C-terminus is an interaction hub

The vast majority of ApaH-like phosphatases consist of just a catalytic domain and do not function in mRNA decapping *in vivo* (6). Kinetoplastida ALPH1 is hence a rare, if not exclusive exception (6). The distinguishing feature of Kinetoplastida ALPH1 within the ApaH-like phosphatase family is the presence of unique N- and C- terminal extensions/domains.

The N-terminal extension is not required for growth in culture, albeit ALPH1ΔN/– cells have a mild proliferation phenotype. This is consistent with low conservation between the Kinetoplastida ALPH1 N-terminal sequences, with 14.6% sequence identity across the lineage, and the lack of N-terminal extensions in the orthologs of *Trypanosoma grayi* and *Leptomonas pyrrhocoris*. The only determined role of the N- terminal domain is in mediating efficient localization to the PP granule.

In contrast, the C- terminal domain is predicted to be partly structured, present in all Kinetoplastida ALPH1 orthologues and better conserved at 31.9% sequence identity across the taxa. This domain is required for efficient localization to starvation stress granules, indicating involvement in interactions with RNA-binding proteins and/or RNA. Further, the C-terminus mediates ALPH1 dimerization and is required for interactions with the 5′-3′ exoribonuclease XRNA and a CMGC-family kinase; both are high-confidence interaction partners. These data indicate a fundamental role for the C-terminus in ALPH1 function and/or regulation. The CMGC-family kinase represents a candidate regulator, and by analogy, phosphorylation contributes to regulation of decapping in opisthokonts (71,72).

### The role of the PP granule remains elusive

ALPH1ΔN/– cells are devoid of ALPH1 localization to the PP granule but viable, excluding a function for the PP-granule as the major location dedicated to the essential process of mRNA decapping, a conclusion supported by the absence of mRNAs or mRNA decay products at the PP. An alternative function for this structure would be to regulate ALPH1 access to mRNA substrates and hence overall mRNA decapping activity. In fact, localization of ALPH1 to the PP is altered when mRNA metabolism is experimentally manipulated: the fraction of ALPH1 at the posterior pole increases when mRNAs are released from polysomes (heat shock, starvation), while blocking RNA synthesis (actinomycin D, sinefungin) or mRNA recruitment to ribosomes (CHX) causes a reduction in PP localization. Hence, cells may attempt to adapt substrate-exposed ALPH1 levels (ALPH1 that is not at the PP) to substrate abundance (non-polysomal mRNAs) in response to environmental cues. However, the proportion of ALPH1 at the PP is extremely low: only 2–3% of ALPH1 molecules are located proximal to the PP in steady state conditions and this is only increased ∼3 fold by heat shock or starvation, with >90% of ALPH1 elsewhere (Figure 8C). These rather moderate changes argue against a function of the PP granule in regulating the concentration of active cytoplasmic ALPH1. It is, however, possible that the fraction of ALPH1 at the PP granule is massively increased in the quiescent, non-dividing life cycle stages of trypanosomes, contributing to the overall reduction in mRNA turnover observed in these stages. This has not yet been investigated. A further possibility is that ALPH1 needs to be recruited to the PP granule in order to be activated and is then released back into the cytoplasm: spatial separation of activation and decapping activity would add a stringent level of regulation. To investigate this, it is in particular important to define which components of the ALPH1 complex localize to the PP independently and which depend on other complex members. We show that XRNA can localize to the posterior pole independently from ALPH1 (Figure 8F).

### A kinetoplastida-specific mRNA decapping complex

Our analyses define an ALPH1 mRNA decapping complex, minimally consisting of ALPH1, XRNA, a CMGC-type kinase, the Tc40-antigen like protein and two RNA binding proteins, Tb927.9.12070 and Tb927.11.3490 (Figure 7C). All components of this core complex share localization to the PP. Several additional proteins are likely additional components, most notably, SCD6 and an RNA helicase (Tb927.4.3890) (Figure 7C–D). These proteins do not localize to the PP, but conditionally, to stress-granules. The RNA helicase shares considerable sequence similarity with Tb927.9.12070 albeit excluding the helicase active site (Supplementary Figure S7B, C), indicating possible mutually exclusive binding to the complex. Evidence for the existence of ALPH1 sub-complexes is also provided by the fact that the ALPH1 IP experiments consistently lacked certain complex members. The likely reason is that the affinity tag was not accessible to the nanobody in a certain ALPH1 complex formation, favouring exclusive purification of another subcomplex (Supplementary Figure S10). However, more experimental work is required to confirm the existence and resolve distribution of ALPH1 sub-complexes.

Importantly, this complex composition is shared with *T. cruzi* (Figure 7B and Supplementary Figure S10B), and, with the exception of the universal XRNA, is Kinetoplastida specific (Figure 7E). The ALPH1 decapping complex appears to be an innovation occurring early within the Metakinetoplastida (Supplementary Table S5). While homologues to ALPH1 and the four core components are absent outside Kinetoplastida, Dcp2 orthologs are present in all remaining eukaryotic lineages, with the exception of Euglenozoa (Figure 7E). Loss of Dcp2, together with the absence of prototypic Dcp2 complex subunits in Discoba and Metamonada, suggests the presence of Dcp2 in the last eukaryote common ancestor, consistent with our earlier study (73). Taken together, this indicates a significant divergence in mRNA degradation mechanisms in Kinetoplastea, as based on the evolution of a novel mRNA decapping complex, relying on a distinct enzyme family for decapping activity, but recruited to the conserved exoribonuclease XRNA/Xrn1.

### Convergent evolution of lineage specific decapping complexes

Decapping flags mRNA molecules for degradation and is a powerful ultimate determinant in mRNA turnover requiring stringent control. For Dcp2, the major decapping enzyme of animals and fungi, multiple regulatory factors share the same Dcp2 binding sites and likely compete (74,75). A variety of regulatory mechanisms involves autoinhibition and interactions with activators, such as Dcp1, Edc1, Edc3 and Pat1 (52,62). Dcp2 and these latter factors are all absent from Kinetoplastida or of sufficient divergence as to be unidentifiable (Figure 7E). Even though there is no known evolutionary relationship between ALPH1 and the canonical nudix domain mRNA decapping enzyme Dcp2, there are some interesting similarities. ApaH/ApaH-like phosphatases and nudix hydrolases have a broad substrate range (76) and both depend on their N- and C- terminal extensions for specificity in mRNA decapping. Many factors specifically interact with the N- and C-termini of Dcp2 (62,63), several of which are shared between the trypanosome ALPH1 complex and Dcp2 (DHH1, SCD6, XRNA/XRN1). mRNA decapping has essentially the same requirements in all eukaryotes, but can be met by different mechanisms and protein families. We propose that the opisthokont Dcp2 complex and the ALPH1 complex in kinetoplastids have converged towards providing the same function, e.g. controlled mRNA decay, and provide a further striking example of convergent evolution in kinetoplastids sitting alongside the kinetochore and nuclear lamina, underscoring the ability of many functions to be achievable with distinct mechanics.

## Supplementary Material

gkad497_Supplemental_Files

## Data Availability

All proteomics data have been deposited at the ProteomeXchange Consortium via the PRIDE partner repository (77) with the data set identifier PXD038550 (all BioID and XRNA affinity capture) and PXD042322 (ALPH1 affinity capture).
