# Supplementary material for: A unique mRNA decapping complex in trypanosomes
Source: Nucleic Acids Res. 2023 Jun 13;51(14):7520–40. doi: 10.1093/nar/gkad497 (PMC10415143; doi:10.1093/nar/gkad497)

FIGURE S1

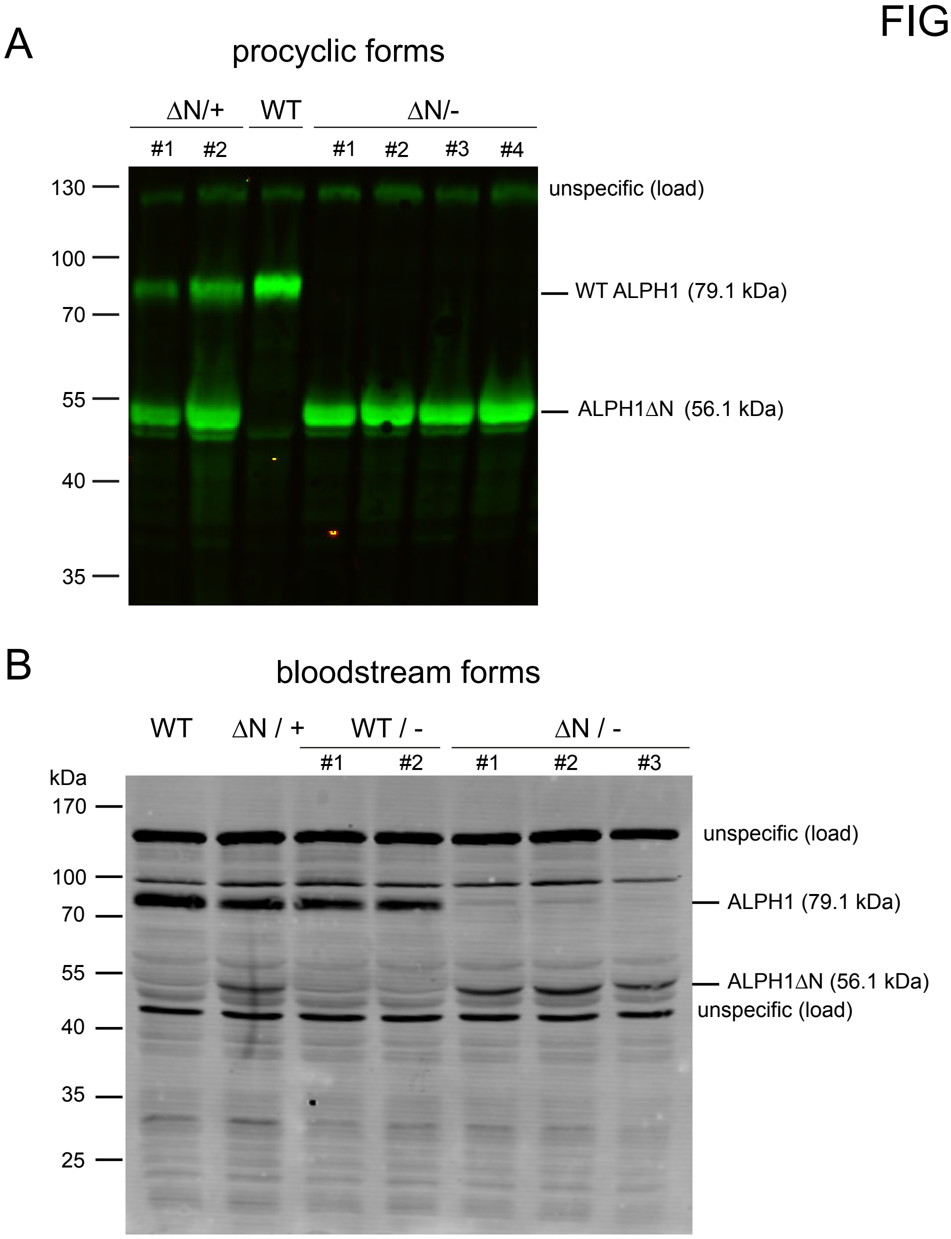

Figure S1: Western blots to control ALPHΔN/- cells

Procyclic trypanosomes **(A)** or MITat1.2 bloodstream form trypanosomes **(B)** were genetically modified by successive transfection with two plasmids to replace one ALPH1 allele with ALPH1ΔN and the second ALPH1 allele by an antibiotica resistance gene (ALPH1ΔN/- cells). Wild type cells (WT) and cells transfected with only one of the plasmids (WT/- or ALPHΔN/+ cells) served as controls. An SDS gel was loaded with cell extracts of clonal cell lines (#) from either wild type cells (WT), ALPHΔN/+ cells, WT/- cells or ALPHΔN/- cells, blotted, and the blot was probed with ALPH1 antiserum.

Note that the ALPH1 antiserum was unstable (and is not available any longer). The western blot with the bloodstream form extract was done at a time, when the antibody started to lose its activity, hence the increase in unspecific signal.

Figure S2

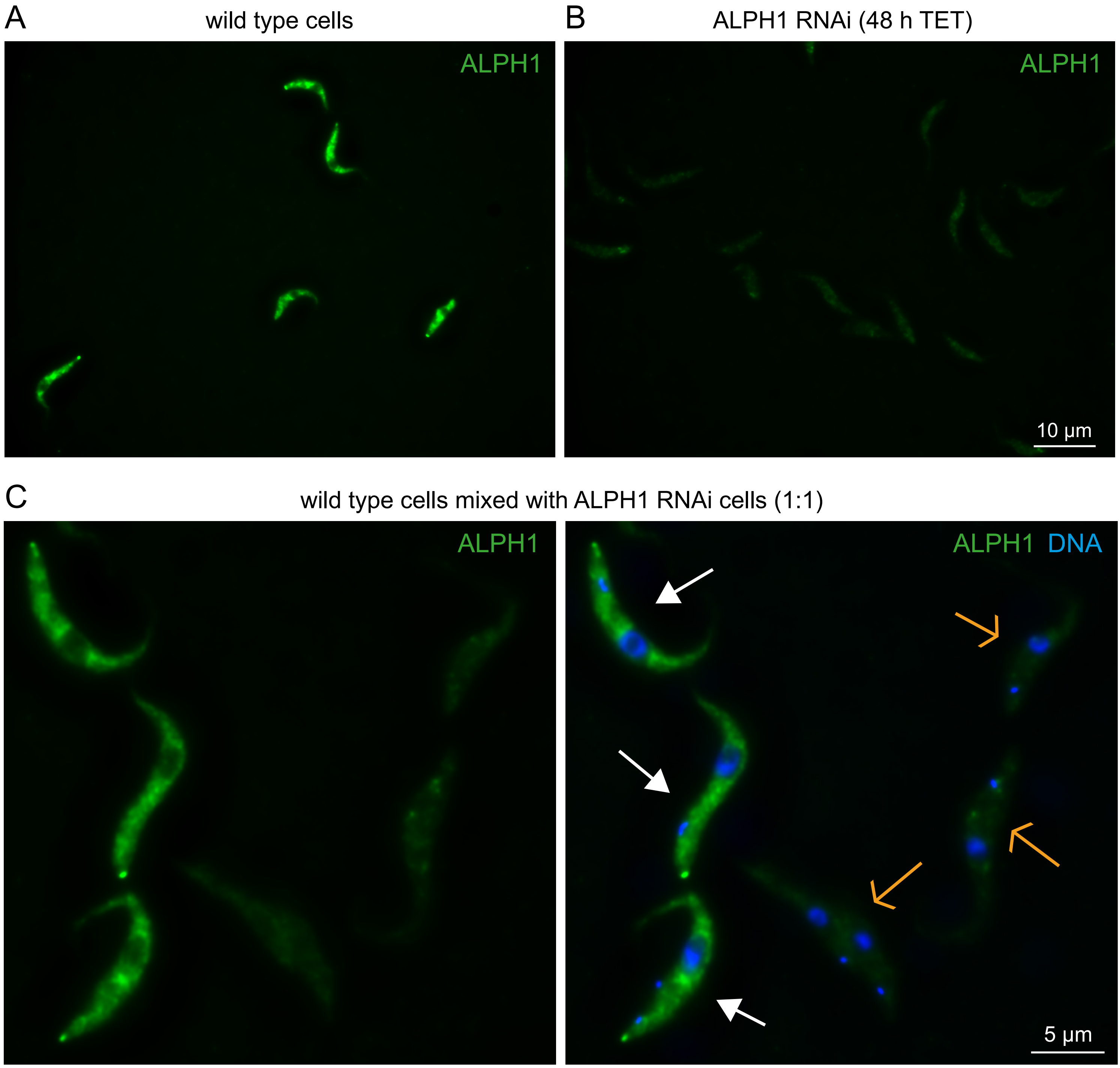

Figure S2: Affinity purified ALPH1 antiserum (1:200) was tested on wild type cells **(A)** and on a previously described ALPH1 RNAi cell line [1] after 48 hours RNAi induction by tetracycline (TET) **(B)**. Both cells were mixed 1:1 after fixation to allow co-probing **(C)**. The white arrow points to wild type cells, the orange arrow to cells depleted for ALPH1 by RNAi.

[1] Kramer, S. (2017). The ApaH-like phosphatase TbALPH1 is the major mRNA decapping enzyme of trypanosomes. PLoS Pathogens 13, e1006456.

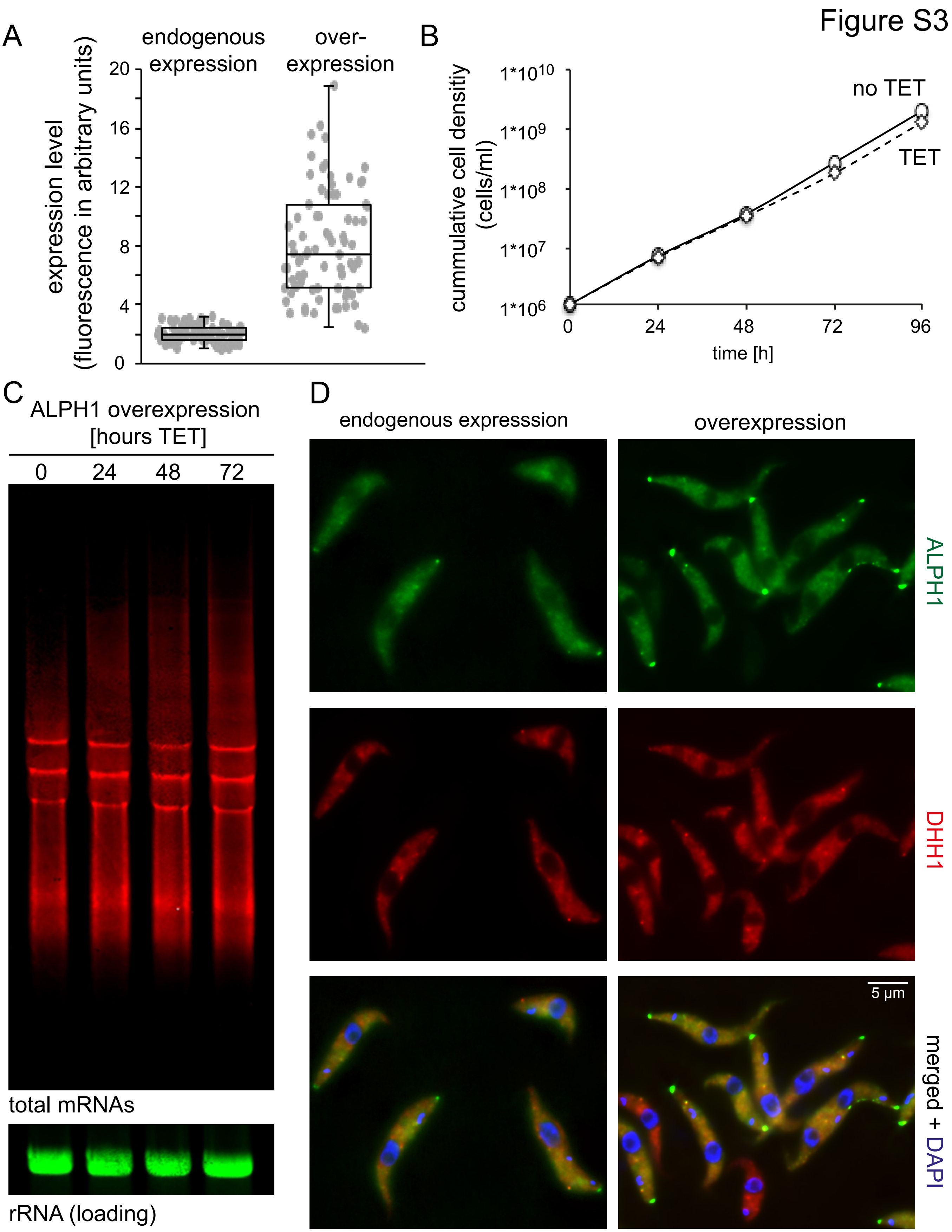

Figure S3: Overexpression of ALPH1

A) The expression levels of ALPH1-eYFP at endogenous expression or inducible overexpression (24 h TET) were compared from microscopy images by quantifying the eYFP fluorescence from projections of deconvolved Z-stack images (method sum slices, background corrected). At least 78 cells were used.

B) Growth curve. Expression of ALPH1-eYFP was induced by tetracyline (TET) and growth was measured in comparison to uninduced cells (no TET).

C) Northern blot with RNA harvested over a time-course of ALPH1 overexpression probed for total mRNA with an oligo antisense to the miniexon (red) and for rRNA (green) as a loading control.

D) Representative microscopy images of cells expressing ALPH1 from the endogenous locus or via the inducible overexpression system. The cells also express mChFP-DHH1 from the endogenous locus as a control.

Figure S4

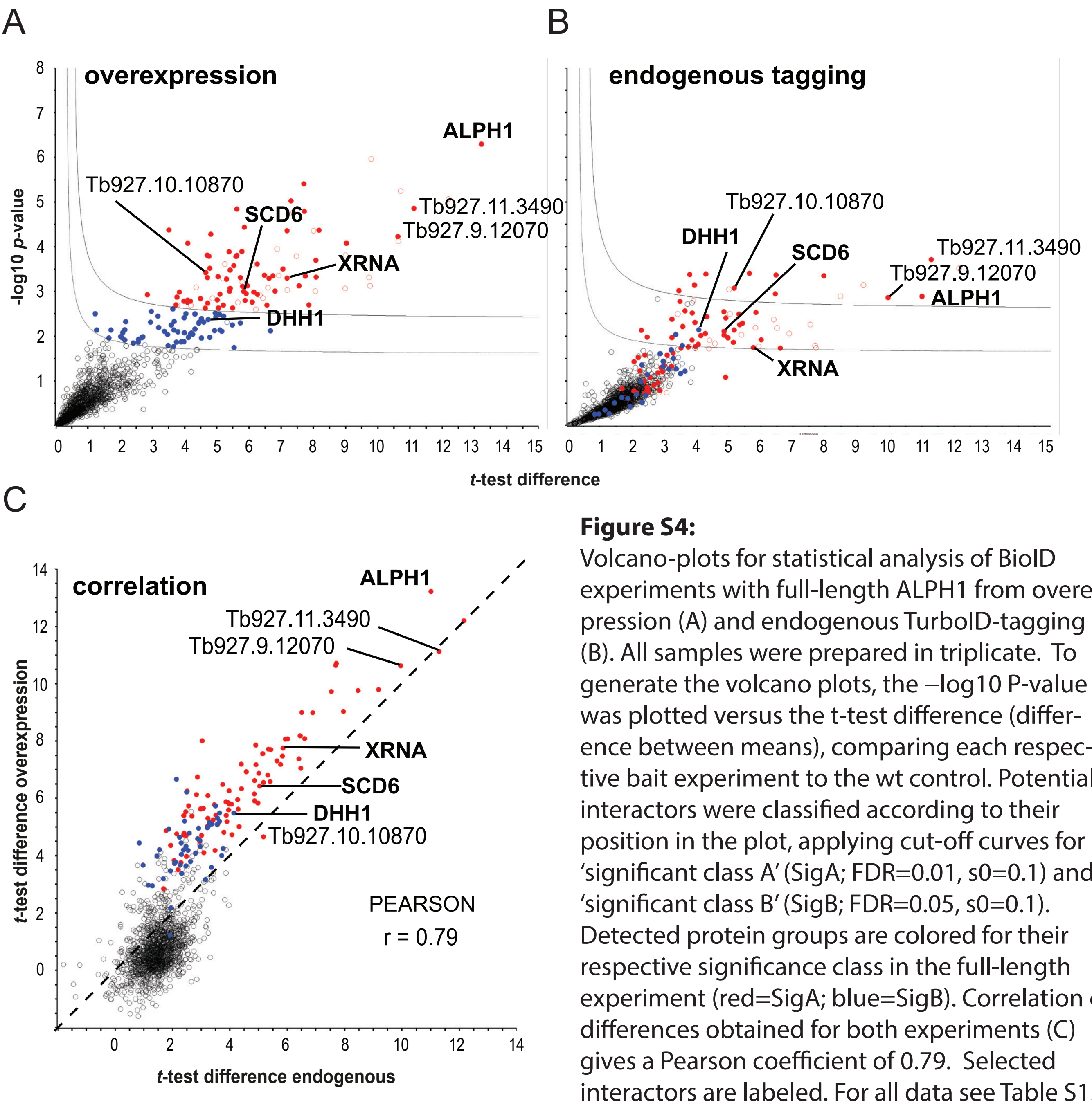

Figure S4:
Volcano-plots for statistical analysis of BioID experiments with full-length ALPH1 from overexpression (A) and endogenous TurboID-tagging (B). All samples were prepared in triplicate. To generate the volcano plots, the –log10 P-value was plotted versus the t-test difference (difference between means), comparing each respective bait experiment to the wt control. Potential interactors were classified according to their position in the plot, applying cut-off curves for 'significant class A' (SigA; FDR=0.01, s0=0.1) and 'significant class B' (SigB; FDR=0.05, s0=0.1). Detected protein groups are colored for their respective significance class in the full-length experiment (red=SigA; blue=SigB). Correlation of differences obtained for both experiments (C) gives a Pearson coefficient of 0.79. Selected interactors are labeled. For all data see Table S1.

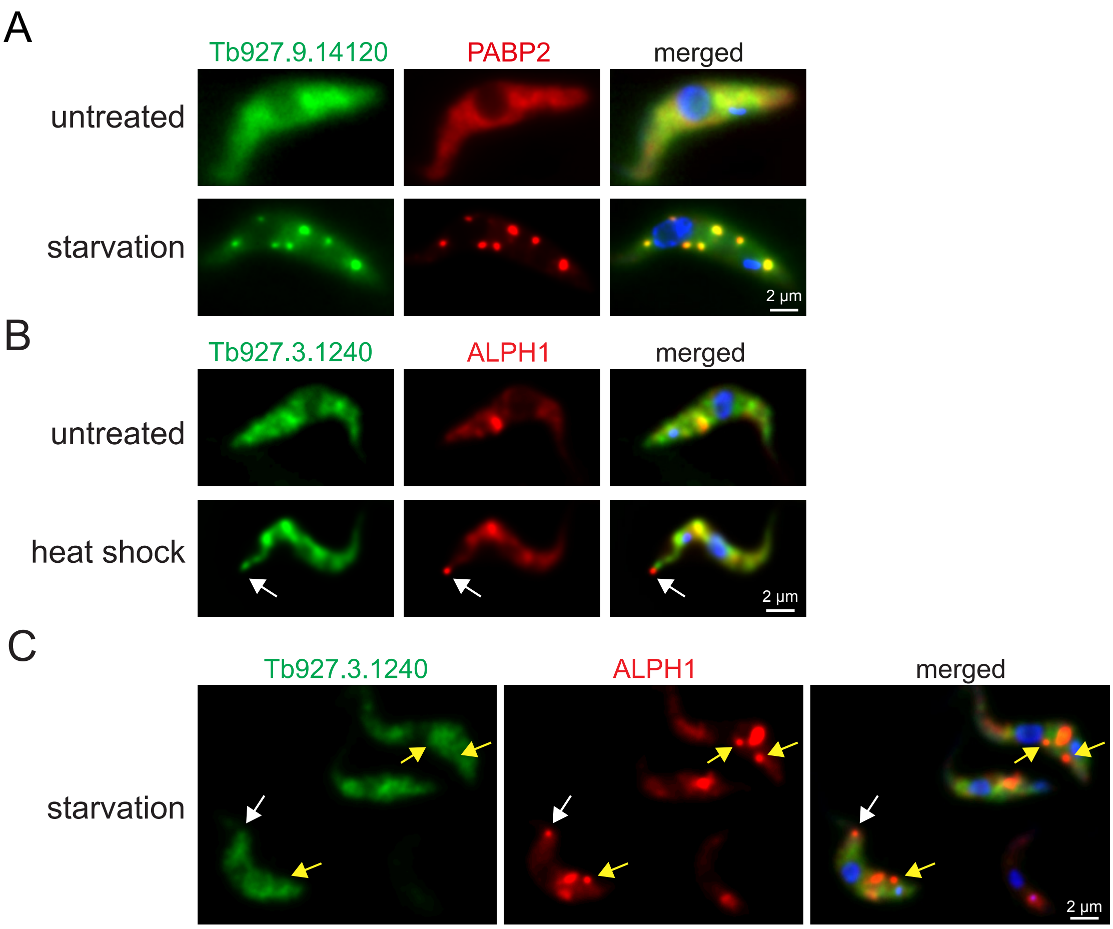

Figure S5: Localisation of Tb927.9.14120 and Tb927.3.1240
A) Tb927.9.14120 was expressed with a C-terminal eYFP-4Ty1 tag from the endogenous locus in a cell line also expressing the stress granule marker protein PABP2-mChFP. A representative image of untreated cells and starved cells (2 hours PBS) is shown as projection of a deconvolved Z-stack (method: sum slices). Tb927.9.14120 colocalises with PABP2 to starvation stress granules.
B and C) Tb927.3.1240 was expressed with a C-terminal eYFP tag from the endogenous locus in a cell line also expressing ALPH1-mChFP. Representative images are shown for untreated, heat-shocked (2 hours 41°C) and starved (2 hours PBS) cells, as single plane images of a deconvolved Z-stack. The posterior pole granule is indicated with a white arrow, stress granules formed by ALPH1 with yellow arrows. There is no evidence for localisation of Tb927.3.1240 to either the posterior pole granule or to starvation stress granules. Note that the expression levels of both proteins are low.

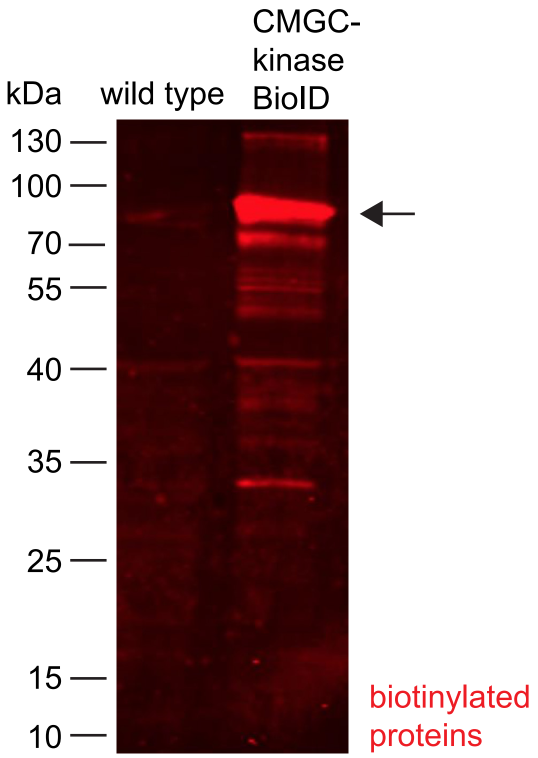

Figure S6: CMGC-type kinase BioID
Westernblot loaded with cell extracts from cells expressing TurboID-HA fusions of the CMGC-type kinase fused to TurboID-HA or from wild type cells (control). The blot was probed with IRDye 800CW streptavidin to detect biotinylated proteins. The arrow points to the protein with the highes biotinylation, which is most likely the bait protein (101.1 kDa).

Figure S7A

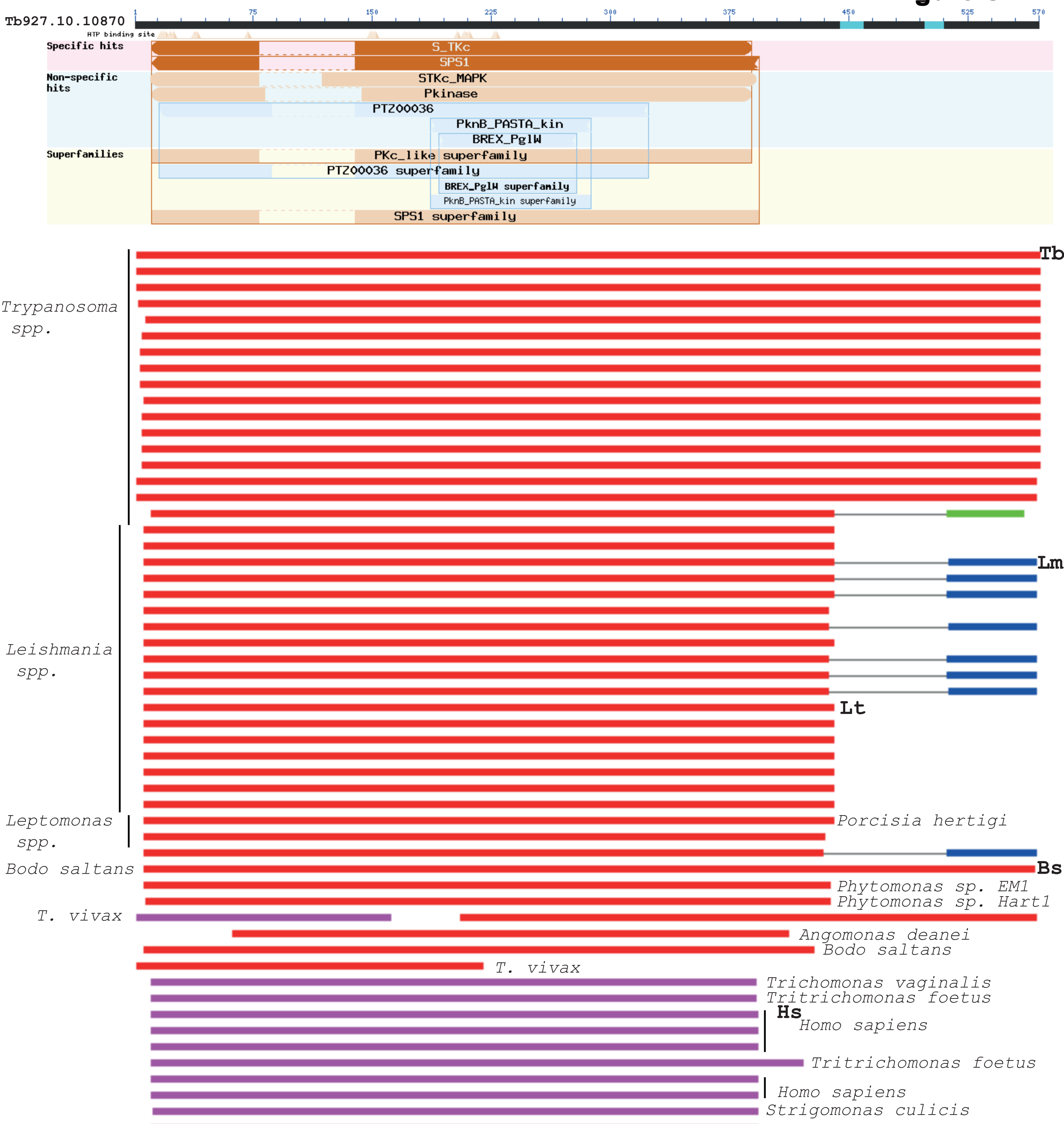
Tb927.10.10870
ATP binding site
Specific hits
S_TKc
SPS1
Non-specific hits
STKc_MAPK
Pkinase
PTZ00036
PknB_PASTA_kin
BREX_PglW
Superfamilies
PKc_like superfamily
PTZ00036 superfamily
BREX_PglW superfamily
PknB_PASTA_kin superfamily
SPS1 superfamily
Tb
Trypanosoma spp.
Lm
Leishmania spp.
Lt
Leptomonas spp.
Porcisia hertigi
Bodo saltans
Bs
Phytomonas sp. EM1
Phytomonas sp. Hart1
T. vivax
Angomonas deanei
Bodo saltans
T. vivax
Trichomonas vaginalis
Tritrichomonas foetus
Hs
Homo sapiens
Tritrichomonas foetus
Homo sapiens
Strigomonas culicis

Figure S7A

```
Hs      ---------RYTQLQYIGEGAYGMVSSAYDHVRKTRVAIKKISPFEHQTYCQRTLREIQI  51
Sc      ---------QYKLVDLIGEGAYGTVCSAIHKPSGIKVAIKKIQPFSKKLFVTRTIREIKL  51
Bs      -----ILAGRYQLVKQIGKGGFGVVEQYRDLFTDALVAIKTIPSRFVDQETKRLVREVDI  55
Tb      MHNVPLLAWRYEHVAVVGKGGFGIVSEHVDRFTGEHVAVKTIPSRFVNQESARLVREIDI  60
Lm      ---MHLLAGRYILVKQIGKGGFGAVEEYTDAITKDNVAIKTIPSRYVNQESRRLVREIDI  57
Lt      -----LLAGRYILIKQIGKGGFGAVEEYTDAITKDNVAIKTIPSRCVNQESRRLVREIDI  55
                 :*  :  :*:*.:* * .  .      **:*.*     .    * :**:.:

Hs      LLRF-R-HENVIGIRDILRAST--------------------------------------  71
Sc      LRYFHE-HENIISILDKVRPVS--------------------------------------  72
Bs      MLHLYGAHPHLMSMLDLFVTRNTAAERQPSIE-FDDEER---QLSEVDAAEIALNQLLIS  111
Tb      MSFLHDAHPHVIGYFDMFATPSGADNVLSQDEGGRD-----GATTLVDARR-------FD  108
Lm      MCFLHEAHPHVIGYFSIFATKGTAIPHHNDSNTFDDPLMSVAQTAENTAFE-------LN  110
Lt      MCFLHEAHPHVIGYFSIFATKGTSIPNHNDSNAFEDLLMSVAQTAENTAFE-------LN  108
        :  :   * :::.  . .

Hs      ------------------------------LEAMRDVYIVQDLMETDLYKLLKSQ-----  96
Sc      ------------------------------IDKLNAVYLVEELMETDLQKVINNQNSGFS  102
Bs      HTQSTTPMPSRDVLLREYALAMQTYANAVHNRSDLNLHIVMPLMKGDLFYFCKHMRSGQ-  170
Tb      AAGPQYPNQSRNDELSKYHDKVLSMVNRLSGEDEFSLHIVMPLMKGDLLYFIKHVSSSGS  168
Lm      VLGEHYAGLSEDERLRLHHEELMAFVAKLTKTDEFNVHIVMPLMKGDLFYFIRLLSSQSS  170
Lt      VLGEHYAGLSEDERLRLHHEELMTFMSKLTKADEFNVHIVMPLMKGDLFYFIRLLSSQSS  168
                                            :::*  **: **  . .

Hs      Q--------LSNDHICYFLYQILRGLKYIHSANVLHRDLKPSNLLINTTC------DLKI  142
Sc      T--------LSDDHVQYFTYQILRALKSIHSAQVIHRDIKPSNLLLNSNC------DLKV  148
Bs      --LMAVSPSFLPQVCVVFAFQVCFGLDYLHKCNVVHRDLKPDNILVWLDLENAYKSTAVI  228
Tb      --KPAMTDEFIELVTVVFAFQICFGLDFLHKCNIVHRDIKPENILVRLHGKDPYKSTALI  226
Lm      VQRLGVTHHFLAQVAVVFAFQICFGLDYLHQCSIIHRDMKPDNVLVRLDITNPYMSTALI  230
Lt      VQRLGVTHQFLAQVAVVFAFQICFGLDYLHQCSIIHRDMKPDNVLVRLDITNPYMSTALI  228
                 :       * :*:  .*. :*...::***:**.*:*:             :

Hs      CDFGLARIAD----PEHDHTGFLTEYVATRWYRAPEIMLNSKGYTKSIDIWSVGCILAEM  198
Sc      CDFGLARCLASSSDSRETLVGFMTEYVATRWYRAPEIMLTFQEYTTAMDIWSCGCILAEM  208
Bs      ADFGLARDAHAT----------ETFYICTRHYRPPEVITNTSKGDTSIDVWSLGCIFFEL  278
Tb      ADFGLARDAQAS----------DTFYVCTRYYRPPEIVTNVSGGEPSIDVWSVGCILFEM  276
Lm      ADMGLARDAQHS----------DTIYICTRYYRPPEVITSVSGGSPKIDIWSLGCIFYEM  280
Lt      ADMGLARDTQHS----------DTIYICTRYYRPPEVITSVSGGSPRIDIWSLGCIFYEM  278
        .*:****                * *:.** ** **:: . .     :*:** ***: *:

Hs      LSNRPIFPG------------KHYLDQLNHILGILGSPSQEDLNCII-NMKARNYLQSLP  245
Sc      VSGKPLFPG------------RDYHHQLWLILEVLGTPSFEDFNQIK-SKRAKEYIANLP  255
Bs      VTARTLFNLPTALNNKGQWEGIKASQQLEVILNTLGTPSKADVERYMPQGNAQSYLIKSR  338
Tb      VTGKALFNVESALNEQGVWDGQLASTQLEVILNIVGTPSHDDIRRFMPVGNAQNYLLRSA  336
Lm      CTGQTLFTMRTALNERGEWDGAKASLQLEVVLNTIGTPAAEDIERYMPSGNAKLYLQRSA  340
Lt      CTGQTLFTMRTALNERGEWDGAKASLQLEVVLNTIGTPSAEDIDRYMPSGNAKLYLQRSA  338
         : : :*                   **  :*  :*:*:  *.       .*: *:

Hs      SKTK-------VAWAKLFPKSDS--KALDLLDRMLTFNPNKRITVEEALAHPYLEQY---  293
Sc      MRPP-------LPWETVWSKTDLNPDMIDLLDKMLQFNPDKRISAAEALRHPYLAMYH--  306
Bs      ARPSVLTDSIRAHWRLGETSTEEQEKWIDLISSCLKFFPQQRPTADQLCAHQLFRDYNVL  398
Tb      PRPSRLVGMMNEQWRLHT-TRDRQQKWIDLISSCLAFFPQQRPTCGDLCRHELFQEYNLF  395
Lm      ARPSQLRQLIEQNWILHT-SDDEKEKWIDLITRCVAFFPEQRPTAQQLCQHQLFRNYNVF  399
Lt      ARPSQLRQLIEQNWILHT-SDDEKEKWIDLITRCVAFFPEQRPTAQKLCQHQLFRKYNVF  397
         :           *     . :   . :**:   : * *::* :  .   *  :  *

Hs      ------------------------------------------------------------  293
Sc      ------------------------------------------------------------  306
Bs      YGENVKQYEPREYAPVTFPP-NSQKSDLKDIVLSLVRRSISRSLDALGARPASS----VG  453
Tb      YGGNVMQYQPKRYQSAEVN---TLKTKNKQSVLHLVRLALQKHSPVVDVSFSDGAKW---  449
Lm      YGSNVKQYAPTPYTSSYCGSSDSTRTENKAAILALVQHALRKTMPPLNEERSDEESSSLN  459
Lt      YGSNVKQYAPTLYTPSYCGSSDSIRAENKAAILTLVQRALRKTMPPMN------------  445
```

Figure S7A

```
Hs     ------------------------------------------------------------  293
Sc     ------------------------------------------------------------  306
Bs     AAEAPQDAATLATTTVRGGLSFPALDDPIL-----FSK------------FSN--MLVAT  494
Tb     -----------KDEDEEGKKSPCAPKERFLDEGELLATPEAVGSDVQDSDMRGEGVTPLE  499
Lm     SSSGSASSGDSESDDERGTAPQPTPINDYVSAAHRCST------S-----LPRDSLQQCG  508
Lt     ------------------------------------------------------------  445

Hs     ------------------------------------------------------------  293
Sc     ------------------------------------------------------------  306
Bs     DGQVDDALDEVLN-----------------------------------------------  507
Tb     GGSEEGELGESIA-FRFL-----------KDSELRRQYDNWGWSGRTRDEVLAKIL----  543
Lm     GCDQNEELGSVAEPFSAFDSTTTSGAPAVPPPAQQQRYALHPFGKNTSKDYCNSFLDDDD  568
Lt     ------------------------------------------------------------  445

Hs     ------------------------------------------------------------  293
Sc     ------------------------------------------------------------  306
Bs     --AMELYTHDANV-----------SRQLRSLLTYFA------------------------  530
Tb     -GDLQRYTHDAVR-----------SEQLRELLRHFSSPR---------------------  570
Lm     EGDVERNRERARDTEDEECLPDFQQQRPSAFMAHFSPASLPDVPLAGAASATAAPAAPAE  628
Lt     ------------------------------------------------------------  445

Hs     ------------------------------------------------------------  293
Sc     ------------------------------------------------------------  306
Bs     ------------------------------------------------------------  530
Tb     ------------------------------------------------------------  570
Lm     PEKRLAHSFSSEESGPVVEDTPPLVPSATLRDRITRRRSSAVQEEEEPKLVSLMPSAFNL  688
Lt     ------------------------------------------------------------  445

Hs     ------------------------------------------------------------  293
Sc     ------------------------------------------------------------  306
Bs     ------------------------------------------------------------  530
Tb     ------------------------------------------------------------  570
Lm     DNYRAGKSLDATVNEELCIPESGLPQAMESMPADDVARLLAAYNMPLEGSNYRTMLSCAQ  748
Lt     ------------------------------------------------------------  445

Hs     ------------------------------------------------------------  293
Sc     ------------------------------------------------------------  306
Bs     ------------------------------------------------------------  530
Tb     ------------------------------------------------------------  570
Lm     SVPPFVMPSDPYHFISQSHIGGGAGAAMLPQQQSFHAAASPNEVNGVPRGGSEGQRGADS  808
Lt     ------------------------------------------------------------  445

Hs     ------------------------------------------------------------  293
Sc     ------------------------------------------------------------  306
Bs     ------------------------------------------------------------  530
Tb     ------------------------------------------------------------  570
Lm     EVRTWAVAPPASRVPPGNGAAAEAGSDEQYDYLAGTDYYIGPTPSNVAQVAAARQPAEKR  868
Lt     ------------------------------------------------------------  445

Hs     ------------------------------------------------------------  293
Sc     ------------------------------------------------------------  306
Bs     ------------------------------------------------------------  530
Tb     ------------------------------------------------------------  570
Lm     GSSPYSLSVADSMPLGQAQQRVLSHASPPPIYLPSTQSSETSLFHQPPLHALRGQSVAGF  928
Lt     ------------------------------------------------------------  445
```

```
Hs      ------------------------------------------------------------   293
Sc      ------------------------------------------------------------   306
Bs      ------------------------------------------------------------   530
Tb      ------------------------------------------------------------   570
Lm      PPIADAELRQRYMSYRHTPRSIQAATSSVLEELGGCTHDAERSSELRQLLNYYTSLEVTT   988
Lt      ------------------------------------------------------------   445

Hs      ----    293
Sc      ----    306
Bs      ----    530
Tb      ----    570
Lm      LYTI    992
Lt      ----    445
```

Figure S7A

Blastp analysis of the CMGC family kinase Tb927.10.10870 searching the NR database including Euglenozoa (taxid:33682), Heterolobosea (taxid:5752), Metamonada (taxid:2611341), Saccharomyces cerevisiae S288C (taxid:559292). Detected domains and distribution of hits (for score > 123) are shown. A multiple sequence alignmentfor selected hits is shown below
(Tb = T*rypanosoma brucei*, Lm= *Leishmania mexicana*, BS = *Bodo saltans*, Sc = *Saccharomyces cerevisiae*, Hs = *Homo sapiens*)

Figure S7B

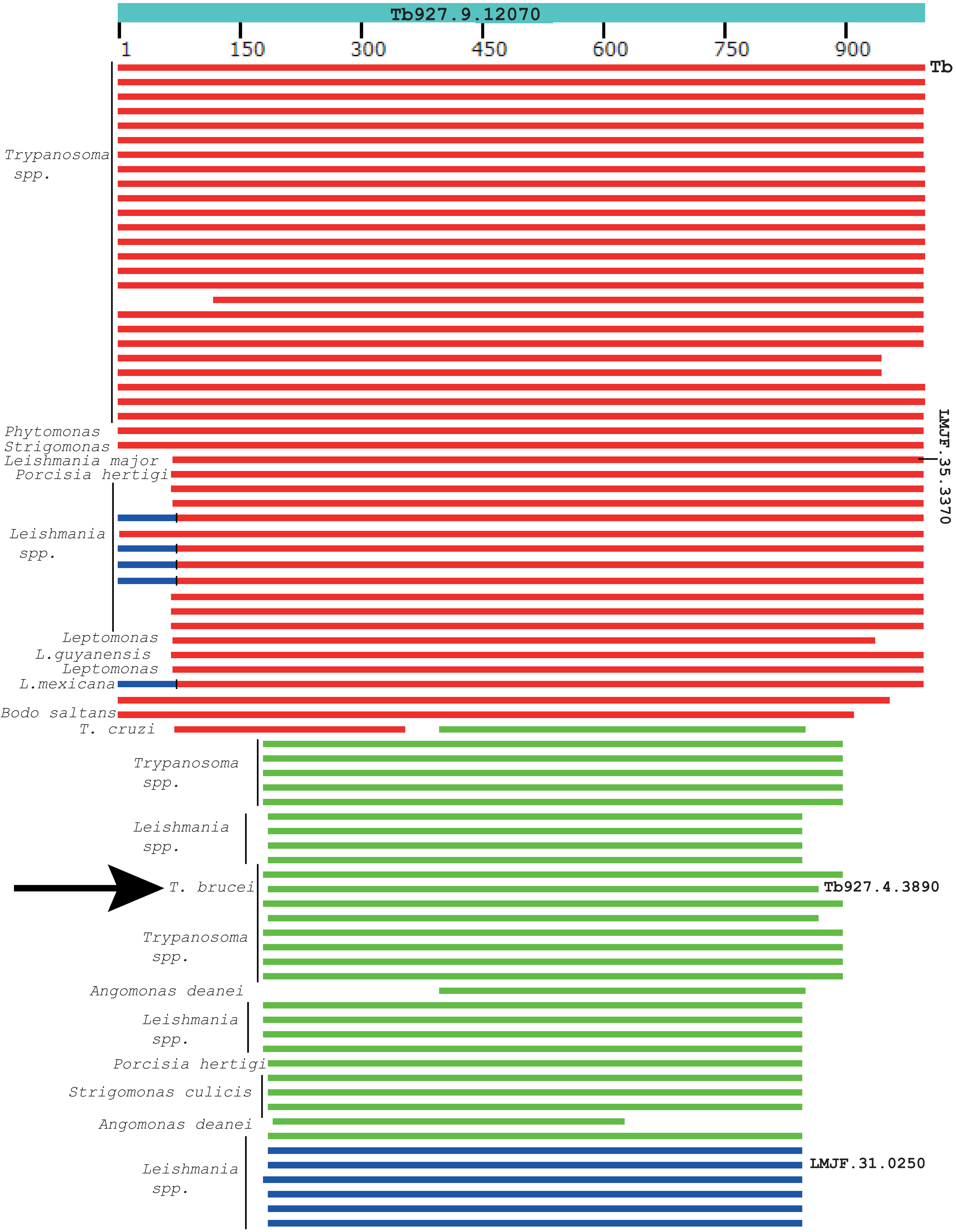
Tb927.9.12070
1
150
300
450
600
750
900
Tb
Trypanosoma spp.
Phytomonas
Strigomonas
Leishmania major
Porcisia hertigi
LMJF.35.3370
Leishmania spp.
Leptomonas
L.guyanensis
Leptomonas
L.mexicana
Bodo saltans
T. cruzi
Trypanosoma spp.
Leishmania spp.
T. brucei
Tb927.4.3890
Trypanosoma spp.
Angomonas deanei
Leishmania spp.
Porcisia hertigi
Strigomonas culicis
Angomonas deanei
Leishmania spp.
LMJF.31.0250

Figure S7B

```
LMJF.31.0250       MPRRSGPQIKRASDKENKDGAAR----------SNDAAKVKVATPA-TVDEQWSTKKQGG   49
Tb927.4.3890       ------------MGKKTRDGPKGGVGNA---IKEMSTTTVTTTTTTSGEGKW-TSSEGG   44
LMJF.35.3370       ------------MPIAPRQGRKGGKISSSPAIASSDSPST-------VSPGSLPFGSAGA   41
Tb927.9.12070      ------------MASAPRLGRKGGKITASPIIPTHALTPGS-----STSPVSLHTPGEGT   43
                                    : *                               .      *

LMJF.31.0250       NVLDWLAQKTDAA-APAPTSRGAVEYLNQTKKPKSYAAAAAPSAAKAKPGTPAEKSAEKA   108
Tb927.4.3890       NLLTWLSQVAPQQQKQNQQSRGAIDYLNKANARKNAKPS------KSSAGRPP--AA---   93
LMJF.35.3370       ALP----------TDPNTTLQFNIEALMSKKRRSPSKAKAAA------------------   73
Tb927.9.12070      TII----------GDPNAAHPFGSDM----------------------------------   59
                    :                      :

LMJF.31.0250       AEKPSIKAPRGAIAREAVRAGKKDNAKAVPAKETPREAPKAETRPAAAPEATAEPRGVPK   168
Tb927.4.3890       --------------------------------------------------ETTSTKPEVPK   104
LMJF.35.3370       ------------------------------------------------------------   73
Tb927.9.12070      ------------------------------------------------------------   59

LMJF.31.0250       PRSKAAPVASPER-KQPDDTVSKLAQLFPAKATGTPST-A------AKPADEPEKVVEAP   220
Tb927.4.3890       PRSLQQPQQQKDQDNAGTNAVEKLAALFPEKQVKEAPA--------------PNAKVGNP   150
LMJF.35.3370       -----RANESSASASSGTEAVK------AASGAGGTSTTAASGSSGASATAGPAETIANP   122
Tb927.9.12070      ----------------------------------------------------------LT   61

LMJF.31.0250       TSATTAVPSVPVPKSSVSAPANAVRPEVPPPRRVRGRRRDDDRLMRAQQRMEAEQESAQR   280
Tb927.4.3890       IAARVAAE---GPKRN----EKERRNETVRAQRHRGEREVK--KERLQRRL-EMMKEAQK   200
LMJF.35.3370       S-----------LVTPPGADSNVFKFDLDAIQ-QVGQGVVFG------------------   152
Tb927.9.12070      V-----------TKKQTSAEANVLQFDVGAIQ-LLGKGVVVG------------------   91
                                        :  : :    :   *.

LMJF.31.0250       RHEEAMRTDAEYKQR--FETMQEMQTAMQMSHAEFFDKYSRQETLLLNHVVTERLDMPVI   338
Tb927.4.3890       FHEEALRTDPEYQKR--FQTFQELQAAQQVAHSTFLDRYAKQGSLNINTIVSERIDIPVI   258
LMJF.35.3370       -GNKTMRTDSHQPESDPAVVYAKLKQLIASQDAYFHGGNA-----AAADVVS---DEVLG   203
Tb927.9.12070      -RAKQARTRTYQPQSDATEVRQRLHGVFSTRRSKVPHRAA-----------------SAG   133
                      :  **     :     .  .::       : .    :

LMJF.31.0250       LDLIEQHDVVFICTDTGSGKSTGVPKALLELSPDTKVVSTQPRRTATVAIA------NRV   392
Tb927.4.3890       LNLIDQHDVVFICTDTGSGKSTSVPKALLELSNDTRVVSTQPRRTATVSIA------TRV   312
LMJF.35.3370       LKEVASHQVVLLQRPYA---ALRLA-VALSQKSKERVLVVVPHYSDVSDIAAA----LRA   255
Tb927.9.12070      LDALQESEVVATRKSQD---TLSLVAALLSADRMANILVVVSKYYELSHVRSSVEAILRE   190
                   *. : . :**          :  :  . *. .   .:: .  :      :        *

LMJF.31.0250       ASLRRESVGEDVGYWIRGDKKGDEQTRLWYMTSYTLLLRILENPAELPFTHIVLDEFHER   452
Tb927.4.3890       AELRREAVGEDVGYWIRGDKCGDVQTRLWYMTSYTLLLHLLNTPLRPPFTHIVLDEFHER   372
LMJF.35.3370       LSPSRGPVGELSG----QSFVGEAGTALWVTDVDTALVYLTSQRGMGPFTHLVLPNLTRM   311
Tb927.9.12070      EGEDTQSVGVLFG----QTFEGSKSTRAWVMTADMALLYMTQLGSPAPFTHVVVPCCVDT   246
                          **   *        *.  *  *       *: : .     ****:*:

LMJF.31.0250       QPDLEVTVALLRLALLNKLSRF-------KLVLMSATLNTEDWEEYFAGLRVATYK---Q   502
Tb927.4.3890       QPDIEVTVALLRLCLLRRTAQF-------KLILMSATLDTEGWEAYFAGLKVATYK---Q   422
LMJF.35.3370       NPLVSYFLWGLRERVYRQSAILDTAAPPLHVIVSVSGAMTERMQQFFAKQTVATP----T   367
Tb927.9.12070      TSSLSCFLKMLGGWMMSNTPRSQ----SVRLVVTTDCSSDGH-----VARTIGVPRVKIL   297
                      :.  :  *   :  .           ::::              .   :..

LMJF.31.0250       SEPEHPIHDYFLEDTCTLLGTDYQAPPQLVSRG--VVDKSTVDKHFYLAQNLILFLNSCS   560
Tb927.4.3890       SEPKHLIHDYFMEDVCTLVGTMYDEPPNIVQRG--VE-YPLMEKHTAIAQALLLYLNTCT   479
LMJF.35.3370       TTPMQPLVEFSYDEANALAGMDVLDVTIDESGKYPGPHRKIIDHSVQMAATLVGRILSCS   427
Tb927.9.12070      DDKVIRLHEFSYNEVCALLGKQTMEMDKDAAGKFPSPPKRLVDYTADVAAELVRYVVT-H   356
                         : ::  ::. :* *                     ::    :*   *:  : :
```

Figure S7B

```
LMJF.31.0250       NPVHSILVFLPGRAQVETMSTWLHTQLYHRVDAVPWHSAVNLSEIEAAMKRNIPGRQKVY   620
Tb927.4.3890       DPQHAILVFLPGRGQVEQMHMWLELNLRKPVDIIPWHSAVDLSVIQEAIKRRGHNRQKIY   539
LMJF.35.3370       TACPQAIYILTGDSR--EMIDALHKE--RLLDTTVYAGRF--PSKEDAG--ATPSKHRVC   479
Tb927.9.12070      TPTAQIFSIFTADVR--EVLTALQGA--KIEDCTVYSTLK--SA---AD--KVETKHRVH   405
                         : :: .  :   :   *.    :  *   :           *       ::::

LMJF.31.0250       LATDIA-EVSITLPDVVYVIDLVLVKRPKISKELPASIQYPPLVTQWISRGSVAQRRGRI   679
Tb927.4.3890       LATDIA-EVSITLPDVVFVIDMVLVKRPKVNKENPATVLHPPLMTQWVSKGNIAQRRGRI   598
LMJF.35.3370       VLHSVVSFTNYNNEDATFVLDMATTRRLAVQNKSEG--FMASSASEWASKMDVEERRQLP   537
Tb927.9.12070      VINHVSHA-LDTENEFTMVLDMGTIRRSSVQHKSES--FIAASTTEWESKAEQAERKSIL   462
                   :   :      .  : . *:*:   :*  :.::  .        ::* *: .  :*:

LMJF.31.0250       GRVQQGFYFCLFPAAQITDLPAYSQPPIENSRIDELSLHCLQVVNNPVAIFSLCHGQPLV   739
Tb927.4.3890       GRVQQGFYFCLLSAEAVAALPEHMHPPIENSRIDELSLHCLQLVSNPVAIFSICHGQPLL   658
LMJF.35.3370       GTKYPGCYIALYPPAADVVLPTTEAPQPTVYEVEDALLQCSRAQ------LPIQRANQ--   589
Tb927.9.12070      GENTTCCYFALFQDDVGASFQDEAQFLPDIFNVENAFVQCARLN------LSVCEVGR--   514
                   *      *:.*      . :           .:::  ::* :        : : .

LMJF.31.0250       EAITSSMNMLTQLGCILDAKDPLSAGERIEEIYSHQNESWSRMIMAAAQAEVMTDIPEYQ   799
Tb927.4.3890       ETIATAMDTLCNLGCILHRKDPLAVGECISEI--HTNSEWSPLVLEEAKNVVFADIDEYE   716
LMJF.35.3370       -------EMVCP----VE-------AESIE-----------QVQHSLSEKCLIANLSDFS   620
Tb927.9.12070      --------LLPS----VP-------RDVVD-----------QVMQKVAEKCMISTPDSLD   544
                            :      :         : :.            :    ::  :::   . .

LMJF.31.0250       YTFIGRILQLIPVSPQPGMLVFFGFLTGLESLMILAAAVTSSLSPF-SINASEGLQR-HF   857
Tb927.4.3890       YTFVGRLLQLIPVSPLPGMLVLYGFFTGLESLTILAAAVTSSLSPF-APNQPEKKRRWRH   775
LMJF.35.3370       LTFTGEIASRLPLEVDLAHFVMNCCTLGHGEVGVIVAGVCALPYRFPTGESAEGFASWKK   680
Tb927.9.12070      ITFLGEIKSRLPVEIDVAYLIMGGCSLGLGEATLVVSAVIALPFRSTAP-PTYTVNRWTE   603
                    ** *.: . :*:.   . :::     *  .  ::.:.* :      :

LMJF.31.0250       NVARAMEETENVMRDFCCG---LRSDIVAVMKATLLFRVEQQRHCNSVETVRHWCLQKHL   914
Tb927.4.3890       DVAGAMEETENSMREMCCG---MRSDILAVMKAVLLFRVQFAKSGEDVTAAKMWCAQHHL   832
LMJF.35.3370       CVIET--------RQEYAGDIANQSDLLAD---VLVFL-EWWRLKARGASTADFVAKLQL   728
Tb927.9.12070      ATQES--------RKRCAGDIALSSDLLAD---AFVFL-EWLRLRTTGAATATFLEAFLV   651
                    .  :        *.  .*     **::*    .::*  :  :      :.  :     :

LMJF.31.0250       SYDKLMAIVDLESHIKYELAEFMPFRSIVEAE---KLLEQLDKLASMVAVMTNVAFVAQS   971
Tb927.4.3890       SYDKLRSILDLEQHIKYELANFIPVRDVPDAT---VLLNQLEKAAAVMLVMTNVAFVSQA   889
LMJF.35.3370       KEDRLEHVRGLITYLRVQISDYAFVDDLEDEATLSAVMQSIKSNASIFTFFEAVALARRL   788
Tb927.9.12070      QEFKFEKIEGLMNHMRDQLMNYAFLDRLDDVDTVNKVAESLRENASTMLMLLSMALSRRA   711
                   .  ::  : .*  ::: :: ::  .  : :      : :.: . *: . .:  :*:  :

LMJF.31.0250       LEVTSEGNSYTNSKEMALG-IFSDLTAVPDIHSPSCLRWQEGDIIIPVQLNLIFDKLLAS   1030
Tb927.4.3890       LEVVSEGNTYQKTKETAVG-IFMDVVAVPDIHSPSCLRWQEGEIIVPVQLSLMGNKMLAS   948
LMJF.35.3370       FFVRDAGSINVKDRAGALVFVRTSKKVVPNTASPSSVVWESGAALVAVDLRNLVSNITCA   848
Tb927.9.12070      AFIRDAGHINEKDRHASMVFVRTSKQLVVHPFIPSGARWETGGIVIPVILKNSTT-ILGG   770
                     : . *    : :  ::  :  .   *  .   **   *: *  :: * *      :  .

LMJF.31.0250       FSTAIASPKQFWISLLLFSQRVQYATFS---DDEGTFHVFALSYGGKERYVEVDEIAGYV   1087
Tb927.4.3890       FSTAISSHKQFWLSLLLLTHKVMYAVFA---DDEGEFYVFSVKYYSTERYLETDAATGSA   1005
LMJF.35.3370       RVSAVN-NTYLFAALLLLYPQVEYSAPVEVPSKGGRVVYFGITCNRQMKRFRISIGEAAQ   907
Tb927.9.12070      MFSLVD-TSLFFASLLLLYPQIEYSRPVT--TERGRVVYFGVACNWQMKRFVVSIDDATQ   827
                     : :   . :: :***:  :: *:       . * .  *.:      : .  .   .

LMJF.31.0250       VLEFRRKLSAICEVLRLTHAHRL-LYEDHFNTLLGSYS-LAPLQDLQREVITALVSIFNN   1145
Tb927.4.3890       ILQFRQMLSSVGQALRLLRDYQH-LYDDEFADVLNDHG-LKPLRDMQCEVVTAMAAFFNN   1063
LMJF.35.3370       ILDFREKWNHAIRYLQVLRTAKKPLSRHKFNFMLKEEDRHFDLEELRAEVQRELQNLVTE   967
Tb927.9.12070      ILDFRENINTAIGCMRALRMLPHPISKTRFAIALKEHDRFFDMERLHRETQRRLHSLAAA   887
                   :*:**.  .     ::  :     :   .*   * . .    :. :: *.   :  :
```

Figure S7B

```
LMJF.31.0250      LE---DM-----------TADEVEHDEDDLD---VVS----------LLSFALPTRAP--   1176
Tb927.4.3890      TE---NI-----------TVDEVEHEEDDLD---EVS----------MISLALPKRG---   1093
LMJF.35.3370      IEVAEHQASFTTHGVHCLAPKQVAGVVDGVDASDVDVSRKFHDGDLWASSFASKTDTPAT   1027
Tb927.9.12070     LNVQEHQGSFETFAKHYTAPKEIIPF-NDVAATDVLLLRRFADGTLWDEQRPSPQSAPAI   946
                   :   .            : .::    :.:                 .

LMJF.31.0250      ------------------------------------------------------------   1176
Tb927.4.3890      ------------------------------------------------------------   1093
LMJF.35.3370      MAAATSITANHVPTTAQSSALT--FPMNAASIDDDKDDEVEVMGELYFRMNGPIIDDDE-   1084
Tb927.9.12070     GSS------------PQKSAVLTPFDTAIPAPYDDDDDDVQIIQNSYFMLHGPLIEDDDD   994


LMJF.31.0250      -  1176
Tb927.4.3890      -  1093
LMJF.35.3370      -  1084
Tb927.9.12070     D  995
```

Figure S7B
Blastp analysis of Tb927.9.12070 searching the NR database including Euglenozoa (taxid:33682), Heterolobosea (taxid:5752), Metamonada (taxid:2611341), *Saccharomyces cerevisiae* S288C (taxid:559292) and *Homo sapiens* (taxid:9606). Detected domains and distribution of hits (for score > 40) are shown. Tb927.9.12070 shares similarity regions (within residues 277-865, Figure S7C) with the RNA helicase Tb927.4.3890 which is also an ALPH1 interactor, but excluded from the PP granule.
A multiple sequence alignment of Tb927.9.12070 and the *L. major* homolog LMJF.35.3370 compared to the RNA helicase Tb927.4.3890 and the respective *L. major* homolog LMJF.31.0250 f is shown below.

Figure S7C

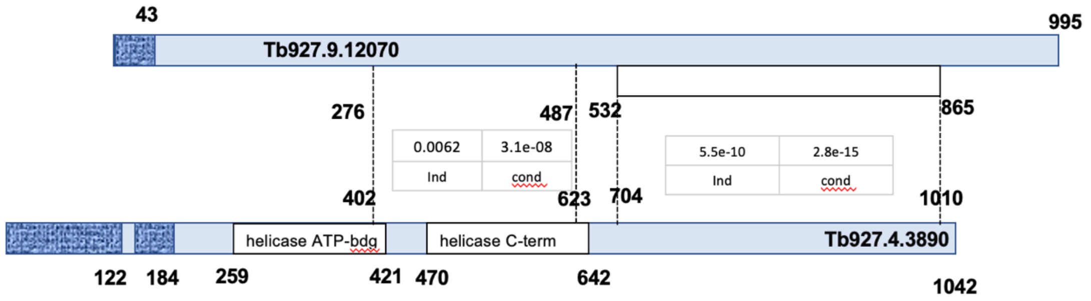

Figure S7C

Similarity regions shared between Tb927.9.12070 and RNA helicase Tb927.4.3890. Independent (Ind) and conditional (cond) e-values are indicated for each region.

Figure S7D

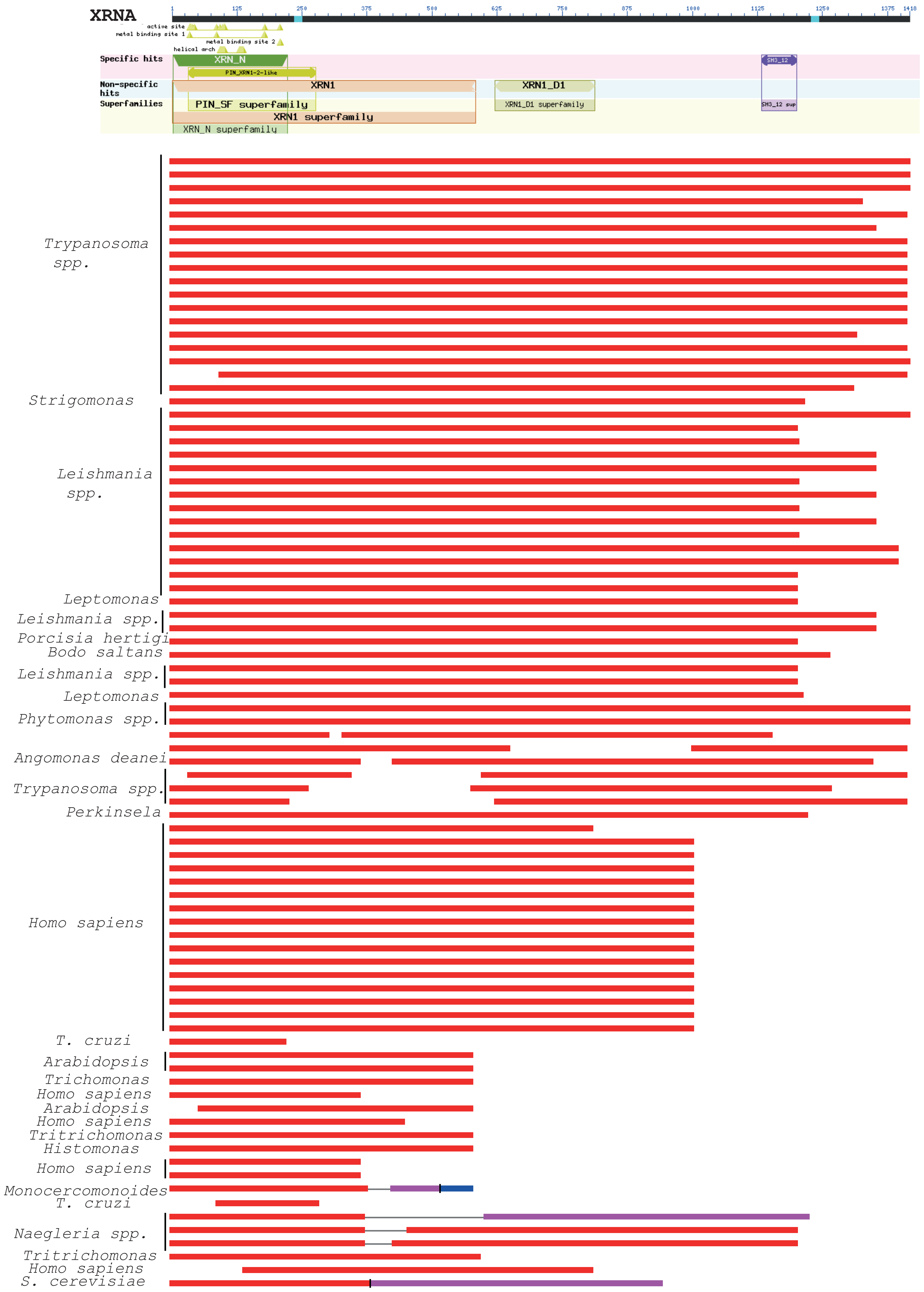
XRNA
1
125
250
375
500
625
750
875
1000
1125
1250
1375
1418
active site
metal binding site 1
metal binding site 2
helical arch
Specific hits
Non-specific hits
Superfamilies
XRN_N
PIN_XRN1-2-like
XRN1
XRN1_D1
SH3_12
PIN_SF superfamily
XRN1 superfamily
XRN1_D1 superfamily
SH3_12 sup
XRN_N superfamily
Trypanosoma spp.
Strigomonas
Leishmania spp.
Leptomonas
Leishmania spp.
Porcisia hertigi
Bodo saltans
Leishmania spp.
Leptomonas
Phytomonas spp.
Angomonas deanei
Trypanosoma spp.
Perkinsela
Homo sapiens
T. cruzi
Arabidopsis
Trichomonas
Homo sapiens
Arabidopsis
Homo sapiens
Tritrichomonas
Histomonas
Homo sapiens
Monocercomonoides
T. cruzi
Naegleria spp.
Tritrichomonas
Homo sapiens
S. cerevisiae

Figure S7D

Blastp analysis of *T. brucei* XRNA (Tb927.7.4900) searching all non-redundant GenBank CDS translations (NR database) including Euglenozoa (taxid:33682), Heterolobosea (taxid:5752), Metamonada (taxid:2611341), Saccharomyces cerevisiae S288C (taxid:559292), Arabidopsis thaliana (taxid:3702) and Homo sapiens (taxid:9606). Detected domains and distribution of hits (for score > 350) are shown.

Figure S7E

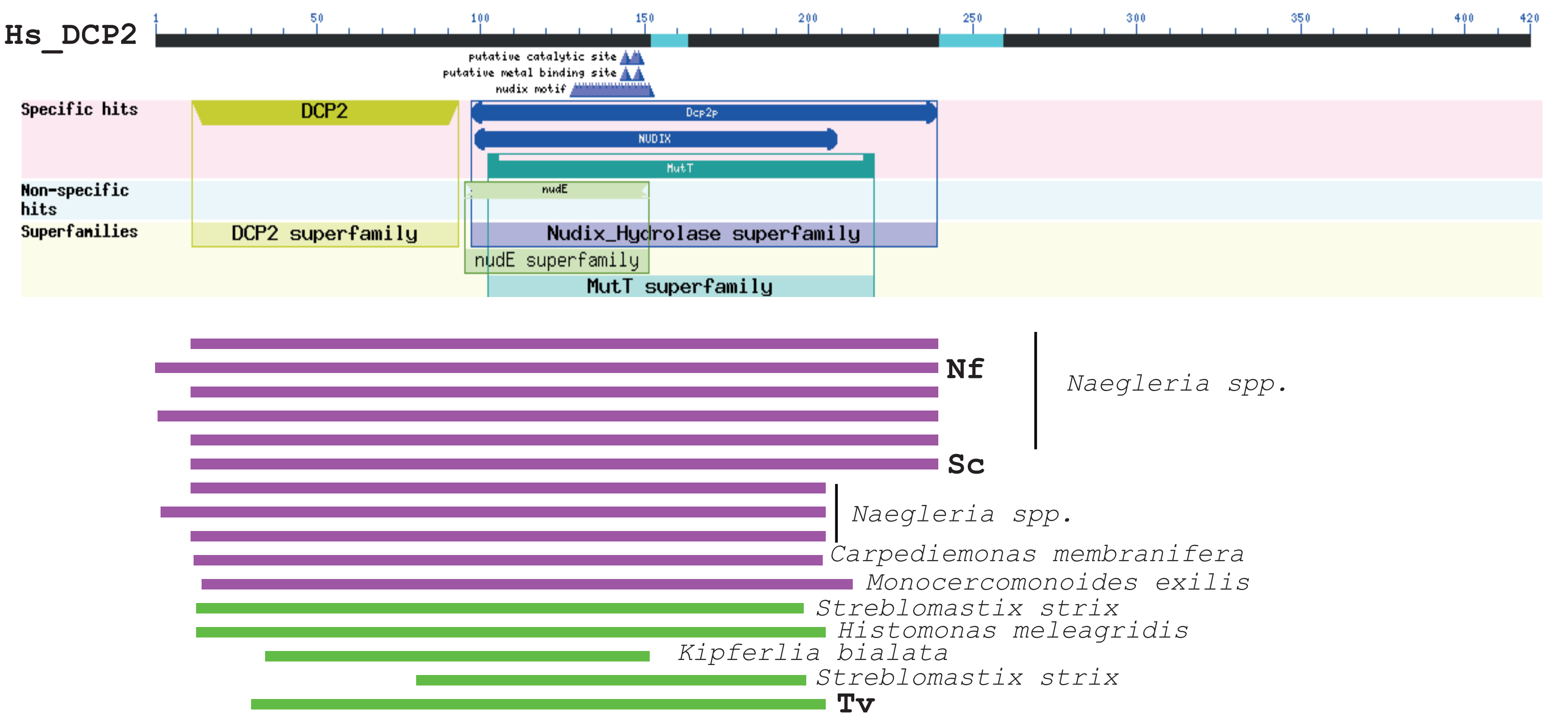


```
Tv_DCP2     ----------------------------------------MNEEQKILEDIAVRFIINQP   20
Sc_DCP2     -----------------------------MSLPLRHALENVTSVDRILEDLLVRFIINCP   31
Nf_DCP2     MSYNNSTNNNHSNPRKSPSRKSPRRGSRDEKMKPNNVANETKSLDDIILELVVKFIMNCP   60
Hs_DCP2     -------------------------------METKRV----EIPGSVLDDLCSRFILHIP   25
                                                          :: ::  :**:: *

Tv_DCP2     YFEEGAKIDLFDLYIQFEQAYWHYIDFYSNKFHKKNQDSIKDKYKTFIKELIQLIPPLQP   80
Sc_DCP2     NEDLSS---VERELFHFEEASWFYTDFIKLMNPTL----PSLKIKSFAQLIIKLCPLVWK   84
Nf_DCP2     EEEHEN---FDRLFFQIEEAFWFYLDFYREQNPSL----PKFNLNQFADQVFLVCPHLQP   113
Hs_DCP2     SEERDN---AIRVCFQIELAHWFYLDFYMQNTPGL----PQCGIRDFAKAVFSHCPFLLP   78
              :           :::* * *.* **             .   . * . ::   * :

Tv_DCP2     FESKILNAMPNFDKFKMSCPVAGIICFNADKSKVIVVRDYSSSHSIGFPKGKISEGESIA   140
Sc_DCP2     WDIRVDEALQQFSKYKKSIPVRGAAIFNENLSKILLVQGTES-DSWSFPRGKISKDENDI   143
Nf_DCP2     FRKSVDEHIQSFISYKTSVPVCGVILLDETLENILLVKGYNS-KSWSFPRGKINQDEEEF   172
Hs_DCP2     QGEDVEKVLDEWKEYKMGVPTYGAIILDETLENVLLVQGYLAKSGWGFPKGKVNKEEAPH   138
                : : : .: .:* . *. *   ::   .::::*:.  :  . .**:**:.: *

Tv_DCP2     QAAIRETIEEIGIDVSPYFRPDQYKCIS-KKKDYHFFYVVGVPENAVMSTIQRNEIYSQQ   199
Sc_DCP2     DCCIREVKEEIGFDLTDYIDDNQFIERNIQGKNYKIFLISGVSEVFNFKPQVRNEIDKIE   203
Nf_DCP2     ACAIREGREEVGFDCSPYLLKDQFLEGRFNEQLVKLFIAPFVPSSTKFLTQTRKEISQIA   232
Hs_DCP2     DCAAREVFEETGFDIKDYICKDDYIELRINDQLARLYIIPGIPKDTKFNPKTRREIRNIE   198
             .. **  ** *:* . *:  :::     : :  :::    : .   :    *.** .

Tv_DCP2     WYPVKELRNELS-----------------MSKIMSKLLSWIEEISNS-------------   229
Sc_DCP2     WFDFKKISKTMY---------KSNIKYYLINSMMRPLSMWLRHQRQIKNEDQLK--SYA-   251
Nf_DCP2     WFNIADIIKK-----------QARGNFWPVKPFLSDLQQWVSKFKTGNRLASIPKKQTPS   281
Hs_DCP2     WFSIEKLPCHRNDMTPKSKLGLAPNKFFMAIPFIRPLRDWLSRRFGDSSDSDNGFSSTGS   258
            *: . .:                         ::  *  *: .

Tv_DCP2     ------------------------------------------------------------   229
Sc_DCP2     ------EEQLKLLLGITKEEQI----DPGRELLNMLHTAVQANSNNNAVSNGQVPSSQEL   301
Nf_DCP2     SPPRTPPKQISRLSTNSHTP----------------------NPFEKHT-----PNL-QL   313
Hs_DCP2     TPAKPTVEKLSRTK-FRHSQQLFPDGSPGDQWVKHRQ-PLQQKPYNNHS-----EMS-DL   310

Tv_Dcp2     ------------------------------------------------------------   229
Sc_DCP2     QHLKEQSGEHNQQKDQ-----------------QSSFSSQQQPSIFPSLSEPFANNKNV   343
Nf_DCP2     F-GRNPSSPHNT------SP--------------AISYTTCDDNSWLT----SFSFTNL-   347
Hs_DCP2     LKGKNQSMRGNGRKQYQDSPNQKKRTNGLQPAKQQNSLMKCEKKLHPRKLQDNFETDAVY   370
```

Figure S7E

```
Tv_Dcp2      ------------------------------------------------------------   229
Sc_DCP2      IPPTMPMANVFMSNPQLFATMNGQPFA-----PFPFMLPLTNNSNSANPIPTPVPPNFNA   398
Nf_DCP2      -----------------V------------------------------------------   348
Hs_DCP2      DLP-------SSSEDQLLEHAEGQPVACNGHCKFPFSSR--------------AFLSFK-   408

Tv_Dcp2      ------------------------------------------------------------   229
Sc_DCP2      PPNPMAFGVPNMHNLSGPAVSQPFSLPPAPLPRDSGYSSSSPGQLLDILNSKKPDSNVQS   458
Nf_DCP2      ------------------------------------------------------------   348
Hs_DCP2      ------F------------------------------DHNAIMKILDL------------   420

Tv_Dcp2      ------------------------------------------------------------   229
Sc_DCP2      SKKPKLKILQRGTDLNSIKQNNNDETAHSNSQALLDLLKKPTSSQKIHASKPDTSFLPND   518
Nf_DCP2      ------------------------------------------------------------   348
Hs_DCP2      ------------------------------------------------------------   420

Tv_Dcp2      ------------------------------------------------------------   229
Sc_DCP2      SVSGIQDAEYEDFESSSDEEVETARDERNSLNVDIGVNVMPSEKDSRRSQKEKPRNDASK   578
Nf_DCP2      ------------------------------------------------------------   348
Hs_DCP2      ------------------------------------------------------------   420

Tv_Dcp2      ------------------------------------------------------------   229
Sc_DCP2      TNLNASAESNSVEWGPGKSSPSTQSKQNSSVGMQNKYRQEIHIGDSDAYEVFESSSDEED   638
Nf_DCP2      ------------------------------------------------------------   348
Hs_DCP2      ------------------------------------------------------------   420

Tv_Dcp2      ------------------------------------------------------------   229
Sc_DCP2      GKKLEELEQTQDNSKLISQDILKENNFQDGEVPHRDMPTESNKSINETVGLSSTTNTVKK   698
Nf_DCP2      ------------------------------------------------------------   348
Hs_DCP2      ------------------------------------------------------------   420

Tv_Dcp2      ------------------------------------------------------------   229
Sc_DCP2      VPKVKILKRGETFASLANDKKAFDSSSNVSSSKDLLQMLRNPISSTVSSNQQSPKSQHLS   758
Nf_DCP2      ------------------------------------------------------------   348
Hs_DCP2      ------------------------------------------------------------   420

Tv_Dcp2      ------------------------------------------------------------   229
Sc_DCP2      GDEEIMMMLKRNSVSKPQNSEENASTSSINDANASELLGMLKQKEKDITAPKQPYNVDSY   818
Nf_DCP2      ------------------------------------------------------------   348
Hs_DCP2      ------------------------------------------------------------   420

Tv_Dcp2      ------------------------------------------------------------   229
Sc_DCP2      SQKNSAKGLLNILKKNDSTGYPRTEGGPSSEMSTSMKRNDATNNQELDKNSTELLNYLKP   878
Nf_DCP2      ------------------------------------------------------------   348
Hs_DCP2      ------------------------------------------------------------   420
```

Figure S7E

```
Tv_Dcp2      ------------------------------------------------------------   229
Sc_DCP2      KPLNDGYENISNKDSSHELLNILHGNKNSSAFNNNVYATDGYSLASDNNENSSNKLLNML   938
Nf_DCP2      ------------------------------------------------------------   348
Hs_DCP2      ------------------------------------------------------------   420

Tv_Dcp2      --------------------------------   229
Sc_DCP2      QNRSSAINEPNFDVRSNGTSGSNELLSILHRK   970
Nf_DCP2      --------------------------------   348
Hs_DCP2      --------------------------------   420
```

Figure S7E
Blastp analysis of human DCP2 searching all non-redundant GenBank CDS translations (NR database) including Euglenozoa (taxid:33682), Heterolobosea (taxid:5752), Metamonada (taxid:2611341), *Saccharomyces cerevisiae* S288C (taxid:559292) and *Homo sapiens* (taxid:9606). Detected domains and distribution of hits (for score > 50) are shown.
A multiple sequence alignment for selected hits is shown below (Tv = *Trichomonas vaginalis*, Nf = *Naegleria fowleri*, Sc = *Saccharomyces cerevisiae*, Hs = *Homo sapiens*)

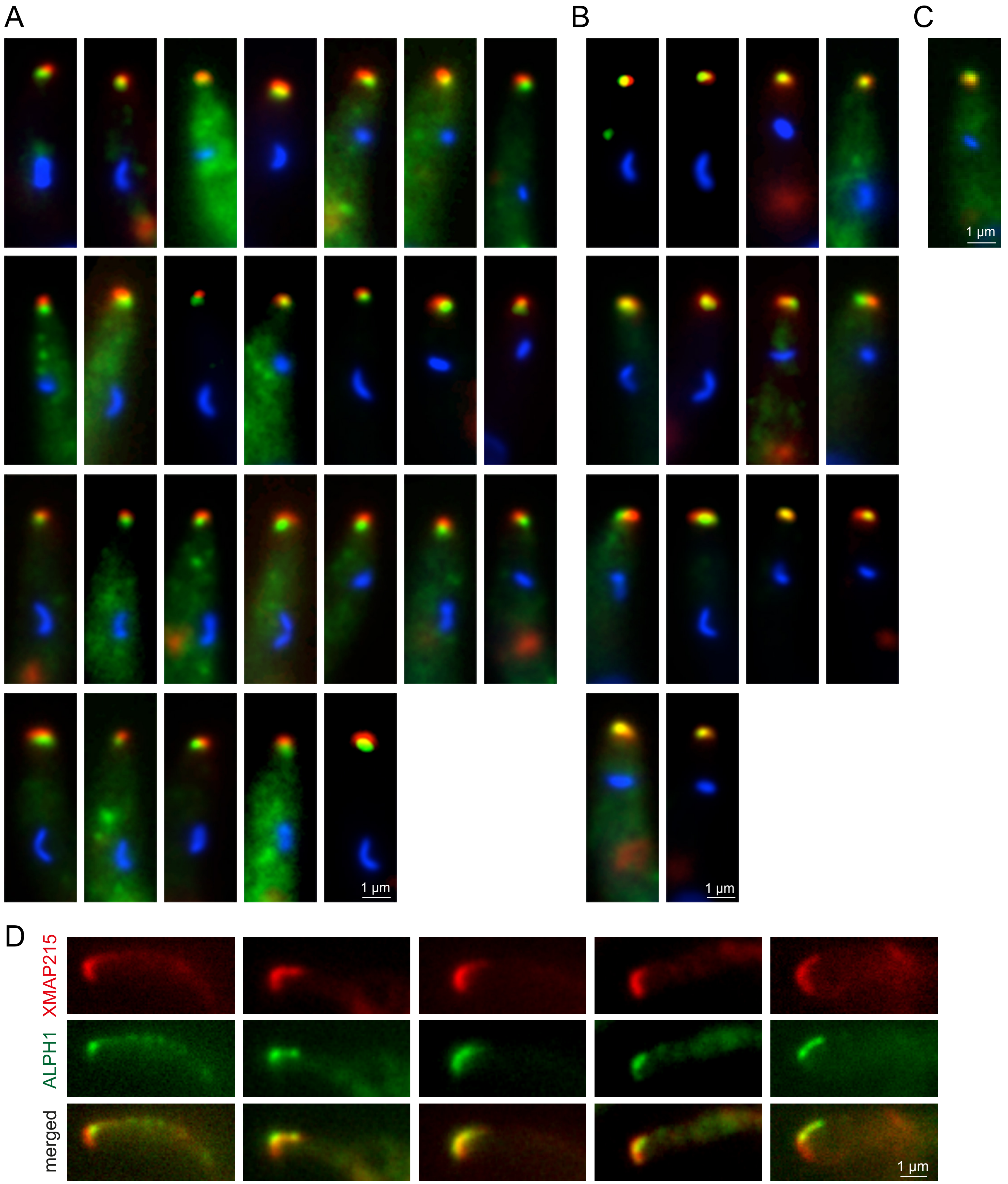

Figure S8: ALPH1 localises anterior to XMAP215

A-C: Microscopic images of 41 randomly chosen cells co-expressing mChFP-XMAP215 (endogenous expression) and ALPH1-eYFP (overexpression). All cells were aligned to have both the kinetoplast and the posterior pole granule in one vertical line and only the relevant section of the cell is shown. The DAPI staining (kinetoplast) is shown as a Z-stack projection image, XMAP215 and ALPH1 are shown as the same single plane from a deconvolved Z-stack that had the strongest fluorescent signal. In most cells, ALPH1 was clearly anterior of XMAP215 (A), in some cells it was adjacent or co-localised (B) and only in one cell it appeared posterior of XMAP215 (C). **(D)** The string-like structure formed by both ALPH1 and XMAP215 during cell division was visualised in life, dividing cells and five representative cells are shown as Z-stack projection of 10 stacks (a 140 nm distance).

Figure S9

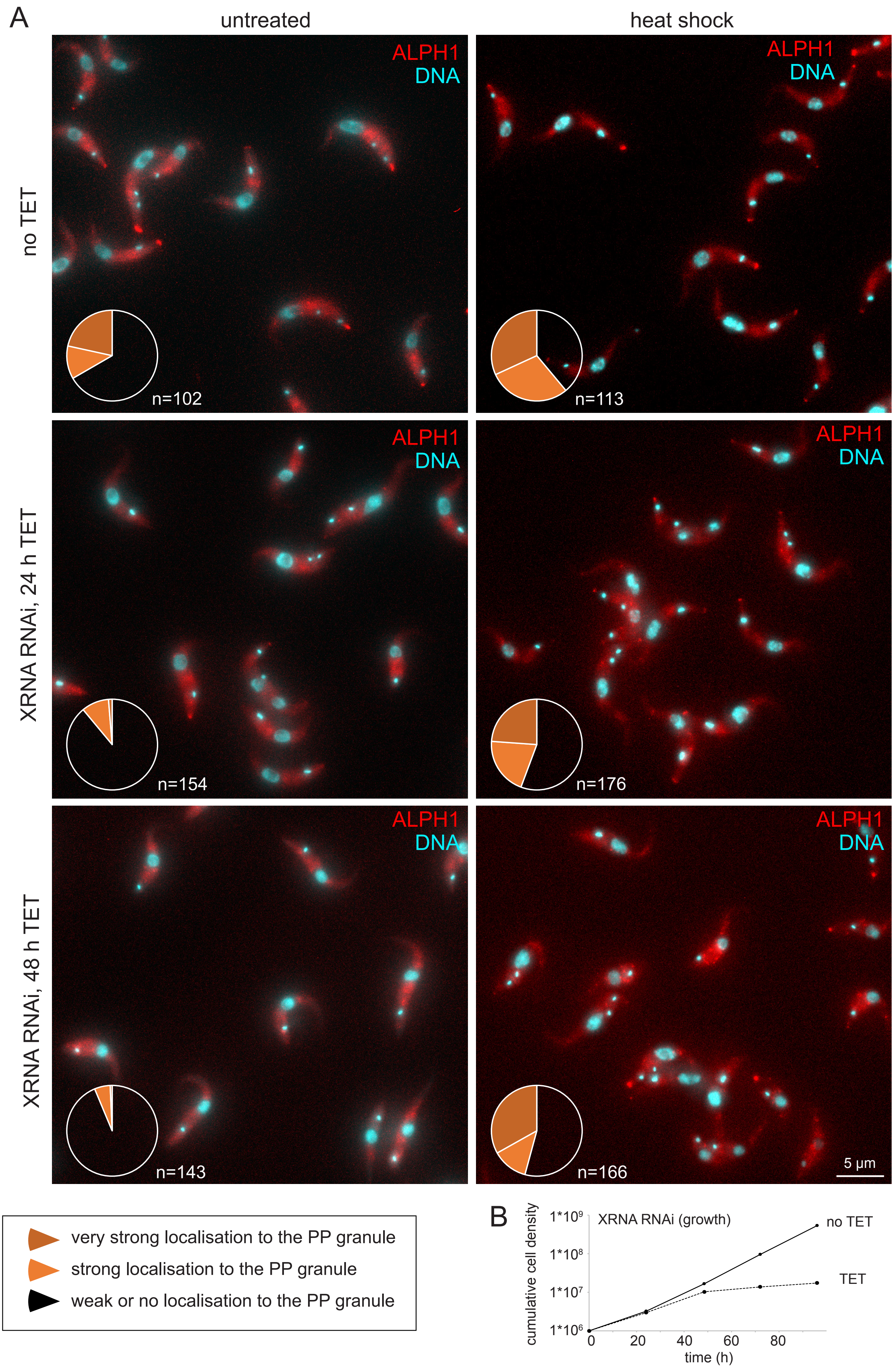

A
untreated
heat shock
ALPH1
DNA
no TET
XRNA RNAi, 24 h TET
XRNA RNAi, 48 h TET
n=102
n=113
n=154
n=176
n=143
n=166
5 µm
very strong localisation to the PP granule
strong localisation to the PP granule
weak or no localisation to the PP granule
B
XRNA RNAi (growth)
cumulative cell density
1*10⁹
1*10⁸
1*10⁷
1*10⁶
no TET
TET
0
20
40
60
80
time (h)

Figure S9: RNAi depletion of XRNA reduced the localistion of ALPH1 to the posterior pole.
A previously described XRNA RNAi cell line [(1)] was used to test, whether reduction in XRNA affects the localisation of ALPH1 to the posterior pole. To visualise ALPH1, one allele of ALPH1 was replaced by an ALPH1 variant with a C-terminal HALO tag. RNAi depletion of XRNA was induced by the addition of tetracycline for 0, 24 and 48 hours and samples of untreated and heat-shocked (2 hours, 41°C) cells were stained with the TMR ligand to visualise ALPH1, fixed and Z-stack images (75 stacks a 140 nm distance) were recorded (see material and methods for details on HALO tag staining). For >100 cells per condition (n), we measured the maximum intensity in a 2x2 µm square at the posterior pole and subtracted the maximum intensity of a control measurement done in a 2x2 µm square anterior of the nucleus of the same cell. We arbitrarily defined any value ≥10 as “very strong localisation to the posterior pole granule”, any value ≥5 but >10 as “strong localisation to the posterior pole granule” and values <5 as “weak or no localisation to the posterior pole granule”. **(A)** For each condition of the experiment, one representative microscopy image (Z-stack projection, maximum intensity method) is shown, as well as the quantifications of posterior pole localisation of ALPH1 of n cells. **(B)** To control the RNAi, a growth curve was done in parallel. The previously observed reduction in growth starting between 24 and 48 hours was confirmed.

[(1)] Kramer S, Piper S, Estevez AM, Carrington M. Polycistronic trypanosome mRNAs are a target for the exosome. Mol Biochem Parasitol. 2016;205: 1–5. doi:10.1016/j.molbiopara.2016.02.009

Figure S10

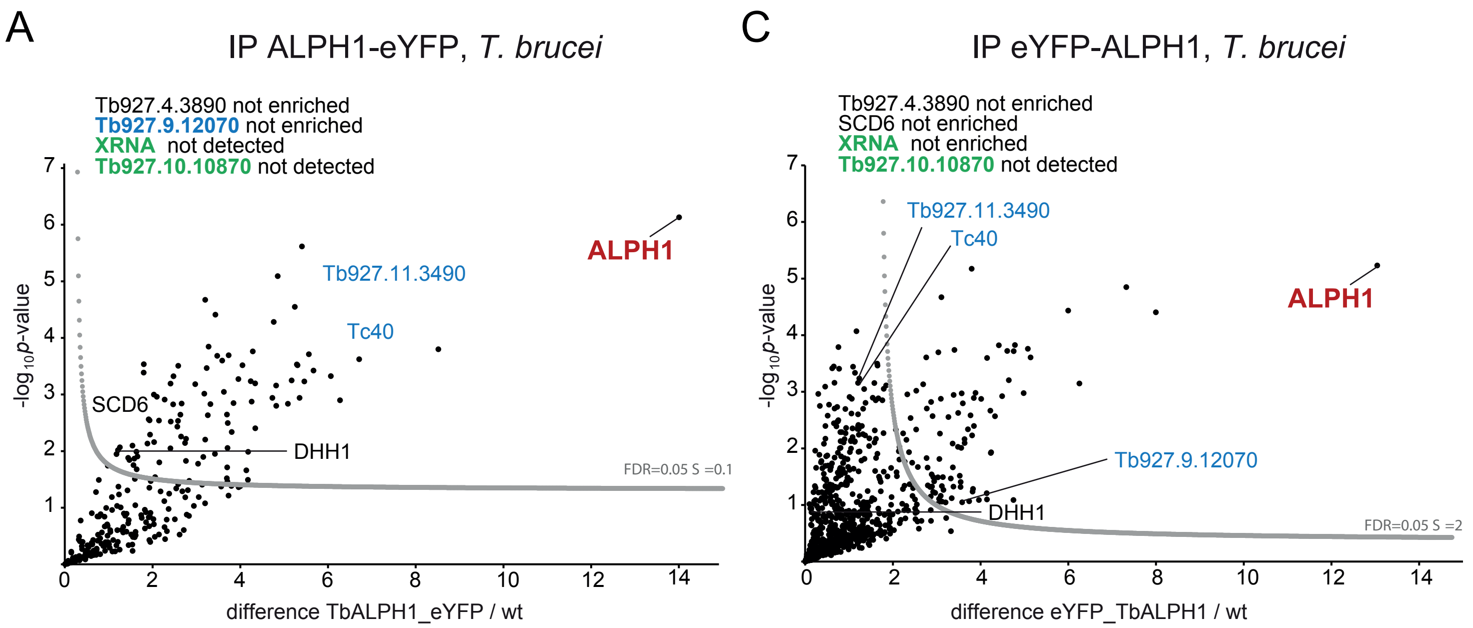

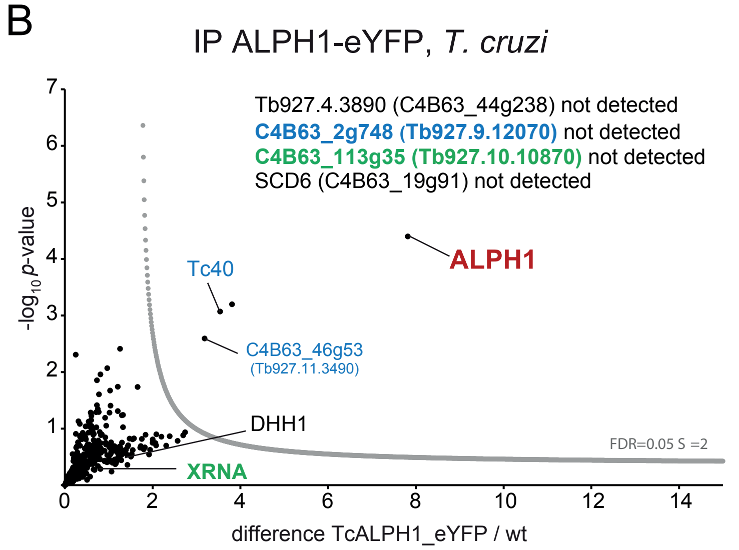

Figure S10. ALPH1 affinity capture delivers a potential sub-complex.
C-terminally tagged ALPH1 was extracted from trypanosome cells via GFP nanobody beads for *T. brucei* **(A)** or *T. cruzi* **(B)** and co-purified proteins were identified by mass spectrometry. The same experiment was repeated with N-terminally tagged TbALPH1 **(C)**. Shown are volcano plots generated by plotting the $-\log_{10}$ P-value versus the t-test difference, comparing the bait experiments (endogenously eYFP tagged ALPH1) to a wild type (wt) control. All proteins that are also in the final stringent list of the ALPH1 BioID are labelled. The statistical cut-offs are indicated by a grey line. The full datasets are shown in Supplementary Table S6. A Venn-diagramm **(D)** details the overlap between significant interactors of the two respective TbALPH1 pulldowns, the TbXRNA pulldown and full-length TbALPH1 TurboID.

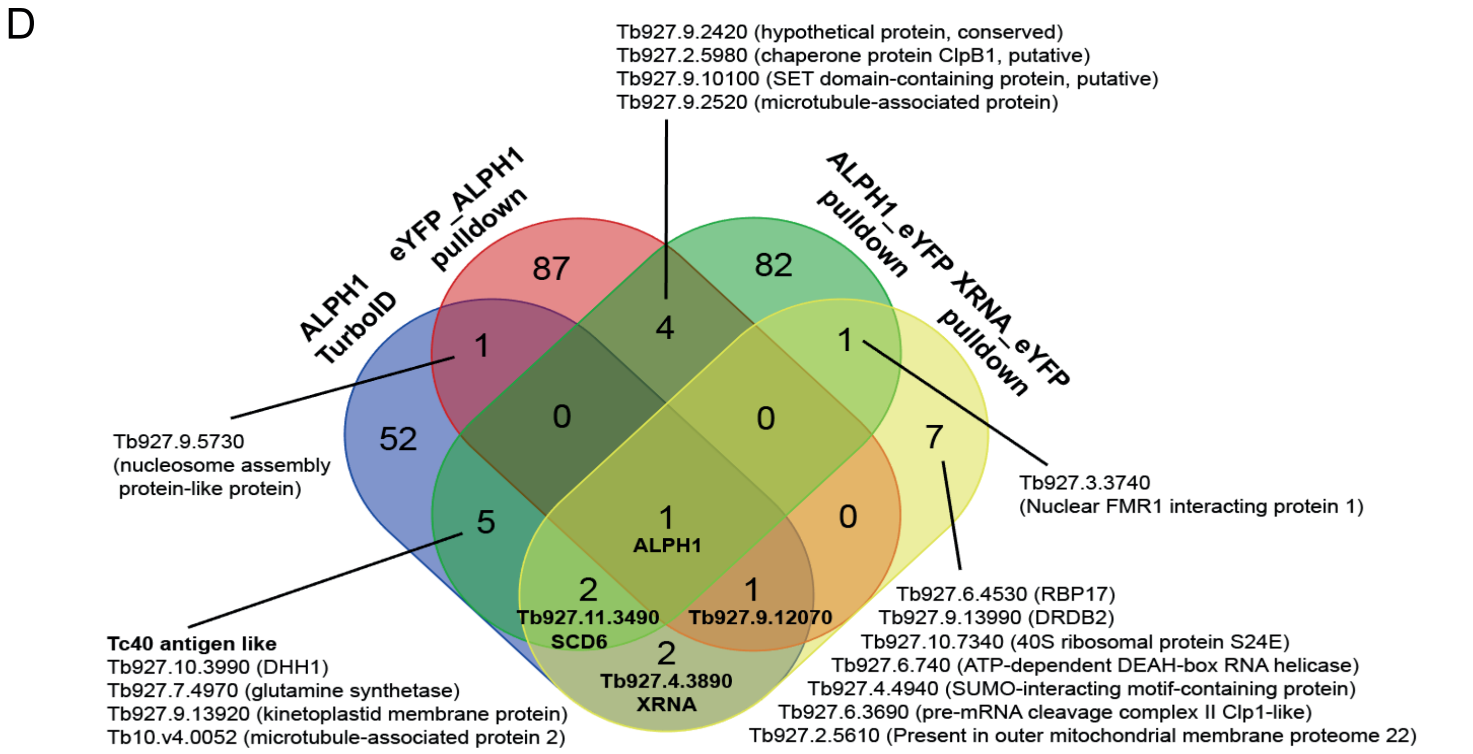

Supplement: gkad497_Supplemental_Files [file gkad497_supplemental_files.zip › SupFig S1-S10.pdf]
